# Novel Drug‐Testing Platform for Vascular Injury‐induced Intimal Hyperplasia Using a Microphysiological System

**DOI:** 10.1002/adhm.202500602

**Published:** 2025-08-11

**Authors:** Ungsig Nam, HyeMi Kim, Jeong Ah Kim, Ki‐Hwan Nam, Kye‐Sung Lee, Hwan Hur, Jessie S. Jeon, Ji Yong Bae

**Affiliations:** ^1^ Center for Scientific Instrumentation Korea Basic Science Institute (KBSI) Daejeon 34133 Republic of Korea; ^2^ Department of Mechanical Engineering Korea Advanced Institute of Science and Technology (KAIST) Daejeon 34141 Republic of Korea; ^3^ Department of Bio‐Analytical Science University of Science and Technology Daejeon 34113 Republic of Korea

**Keywords:** diphenyleneiodonium, intimal hyperplasia, microphysiological system, quercetin, vascular injury

## Abstract

Drug‐coated balloons (DCBs) and drug‐eluting stents (DESs) with antiproliferative drugs have been developed to prevent restenosis. However, some patients who undergo DCB or DES procedures still experience restenosis. Therefore, it is essential to explore therapeutic agents for DCBs and DESs. Furthermore, alternative platforms addressing current experimental model limitations are necessary for disease research. Here, a 3D model of vascular injury‐induced intimal hyperplasia is developed by using a microphysiological system (MPS). This model effectively replicated the endothelial denudation, proliferation, and migration of vascular smooth muscle cells (VSMCs), and vascular inflammation associated with the disease. Using this disease model, it is shown that antiproliferative drugs suppress VSMC proliferation but worsen endothelial denudation. In addition, potential alternatives are investigated to antiproliferative drugs and tested various drugs aimed at reducing inflammation. Partial improvements are found in VSMCs treated with DPI and in the endothelium treated with quercetin. When diphenyleneiodonium (DPI) and quercetin are combined, VSMC proliferation, migration, and vascular inflammation are reduced without impairing re‐endothelialization. This disease model shows promise; this study may offer new treatment insights for DCBs and DESs.

## Graphical abstract

This study develops a 3D vascular injury model using a microphysiological system that mimics key features of intimal hyperplasia. Antiproliferative drugs reduced VSMC proliferation but worsened endothelialdenudation. A combination of diphenyleneiodonium and quercetin effectively reduced proliferation and migration of VSMC and inflammation while preserving re‐endothelialization.

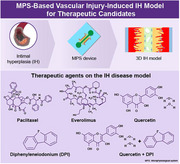

## 1 Introduction

Vascular intervention for occluded arteries is conducted to restore blood flow, and it includes widening the lumen of the artery (balloon angioplasty and stent implantation) and bypass grafting using blood vessels from other tissues.^[^ ^1^ ^]^ However, patients with in‐stent restenosis accounted for 10.6% from 2009 to 2017, and the 10‐year failure of coronary artery bypass graft surgery rate, which is defined as complete occlusion, is 40%−50%.^[^ ^2^, ^3^ ^]^ Intimal hyperplasia (IH), also called neointimal hyperplasia, has been considered one of the major causes of both restenosis and graft failure.^[^ ^3^, ^4^ ^]^ In the development of IH, the endothelium of the artery is injured during vascular intervention, which causes aggregation and accumulation of platelets and immune cells in the injured area.^[^ ^1^, ^5^, ^6^ ^]^ The cells secrete various biomolecules, including inflammatory cytokines and growth factors, to restore the damaged endothelium, but due to these biomolecules, the vascular smooth muscle cells (VSMCs) of the artery simultaneously proliferate, migrate, and synthesize extracellular matrix (ECM), which results in thickening of the arterial intima.^[^ ^1^, ^5^, ^6^ ^]^ To prevent the development of IH after the medical procedure, balloons or stents coated with antiproliferative drugs have been used, but adverse events, including restenosis, have been still reported.^[^ ^2^, ^7^, ^8^, ^9^ ^]^ The one presumed cause of partial success in drug‐coated balloons (DCBs) and drug‐eluting stents (DESs) is that antiproliferative drugs not only inhibit the proliferation and migration of vascular smooth muscle cells (VSMCs) but also delay recovery of endothelium.^[^ ^10^, ^11^, ^12^ ^]^ Therefore, it is essential to develop and explore therapeutic agents that inhibit vascular injury‐induced IH and overcome limitation of antiproliferative drugs used in DCBs and DESs.

In studies of vascular injury‐induced IH to understand its pathophysiology and find therapeutic approaches, rodent models have been widely used due to their ease of breeding and disease induction through genetic modification.^[^ ^13^ ^]^ However, rodents require specialized handling because their arterial diameters are small.^[^ ^13^ ^]^ In contrast, disease models using non‐rodent species such as rabbits, pigs, dogs, and primates are easier to handle due to their relatively larger arteries, but they face limitations in genetic modification and require increased time and cost for breeding.^[^ ^13^ ^]^ Notably, all animal models have the disadvantage of genetic differences from humans, which results in disparate disease environments that make it challenging to directly apply findings in animal models to human cases.^[^ ^13^, ^14^ ^]^ In addition, animal experimental models are always associated with ethical concerns. Most studies on in vitro IH models involve culturing VSMCs and endothelial cells (ECs) separately in a 2D environment, which creates a considerable difference from the in vivo environment where both VSMCs and ECs coexist in a 3D structure.^[^ ^15^ ^]^ Therefore, there is a need for alternative platforms that can complement and replace existing in vitro and animal models for the study of vascular injury‐induced IH.

Microphysiological system (MPS)‐based experimental models, which have been actively studied, can overcome the shortcomings of animal models by using human‐derived cells and controllable stimulation, as well as the shortcomings of in vitro 2D experimental models through the creation of a 3D culture environment with more than two types of cells using the ECM.^[^ ^15^, ^16^ ^]^ The MPS‐based model could be the most optimal drug‐testing platform for vascular injury‐induced IH because drugs coated on DCB and DES are directly delivered without drug metabolism. There have been worthy studies on MPS‐based 3D arterial disease models, which recapitulated atherosclerosis, hyperlipidemia, diabetes mellitus, drug‐induced injury, geometrical stenosis, and Hutchinson‐Gilford progeria syndrome.^[^ ^17^, ^18^, ^19^, ^20^, ^21^, ^22^, ^23^, ^24^, ^25^ ^]^ Although MPS has been utilized in a study on DES, its application has been limited to 2D EC cultures to evaluate stent coating materials.^[^ ^26^ ^]^ In other words, no appropriate 3D model for vascular injury–induced IH using MPS has been established to date. Therefore, it is crucial to develop an MPS‐based 3D model of vascular injury–induced IH, specifically aimed at assessing drug candidates used in DCBs and DESs.

Herein, we proposed a novel drug‐testing platform for vascular injury‐induced IH using an MPS. To achieve our goal, we first investigated the optimal culture conditions for the proliferation and migration of VSMCs cultured in an MPS. Next, ECs and VSMCs were co‐cultured to establish the condition of the IH model, reflecting the representative characteristics of IH, and the differences in characteristics between the normal and IH arterial models were analyzed. To verify the potential of our IH model as a drug‐testing platform, we investigated its response to antiproliferative drugs. In addition, we attempted to treat various therapeutic agents to alleviate pathological changes in the disease and determined the optimal drug condition for our disease model. Lastly, we analyzed the relative cytokine secretion and gene expression levels among the control, IH, and optimal drug conditions. This 3D model and optimal drug condition are expected to represent insights for therapeutic strategies for IH.

## 2 Results

### 2.1 Investigation of Condition for VSMC Proliferation and Migration

**Figure 1:**
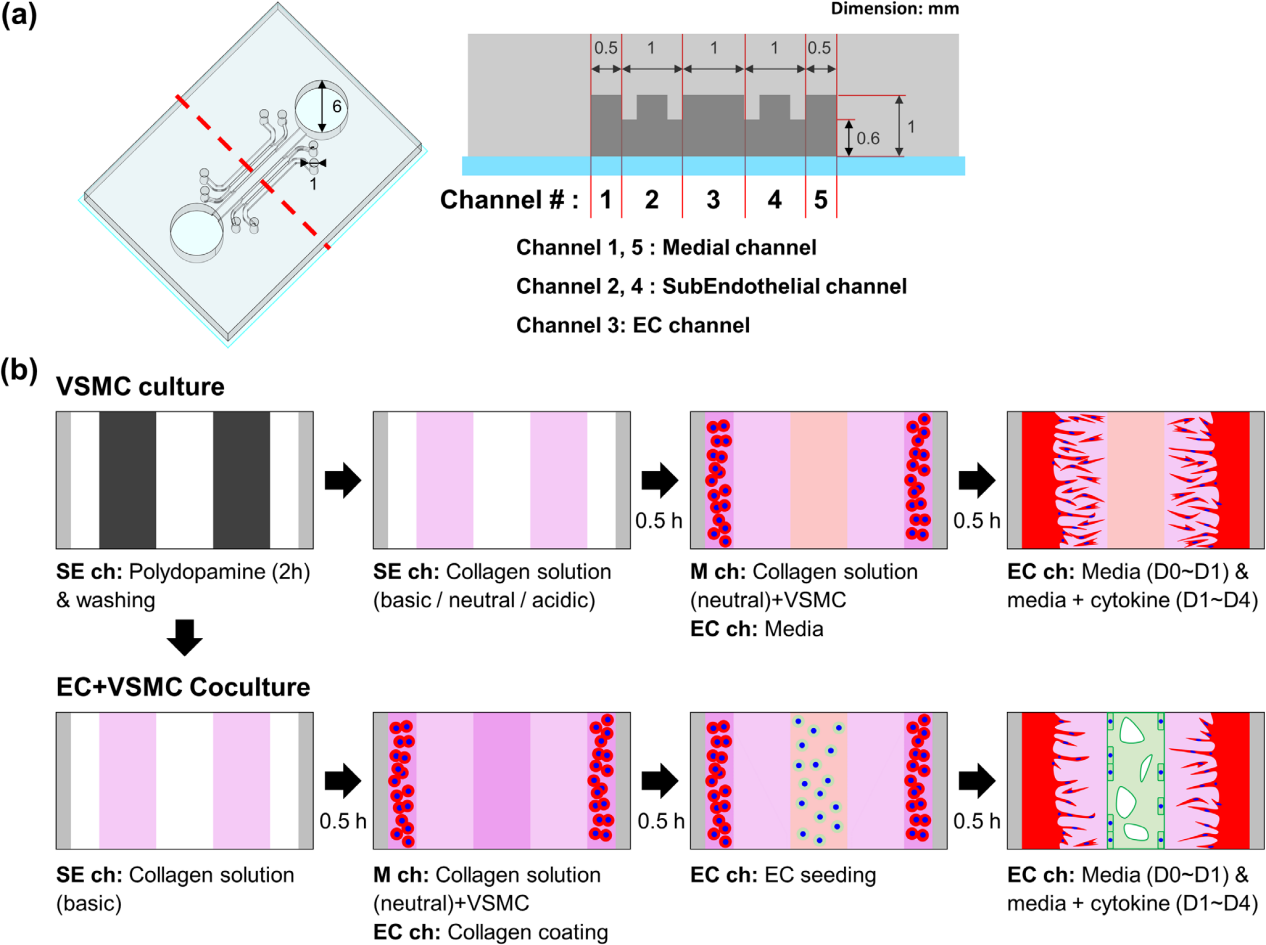
a) Design of microphysiological device composed of five channels. b) 3D culture of arterial cells in a microphysiological system. SE: subendothelial, M: medial, ch: channel.

**Figure 2:**
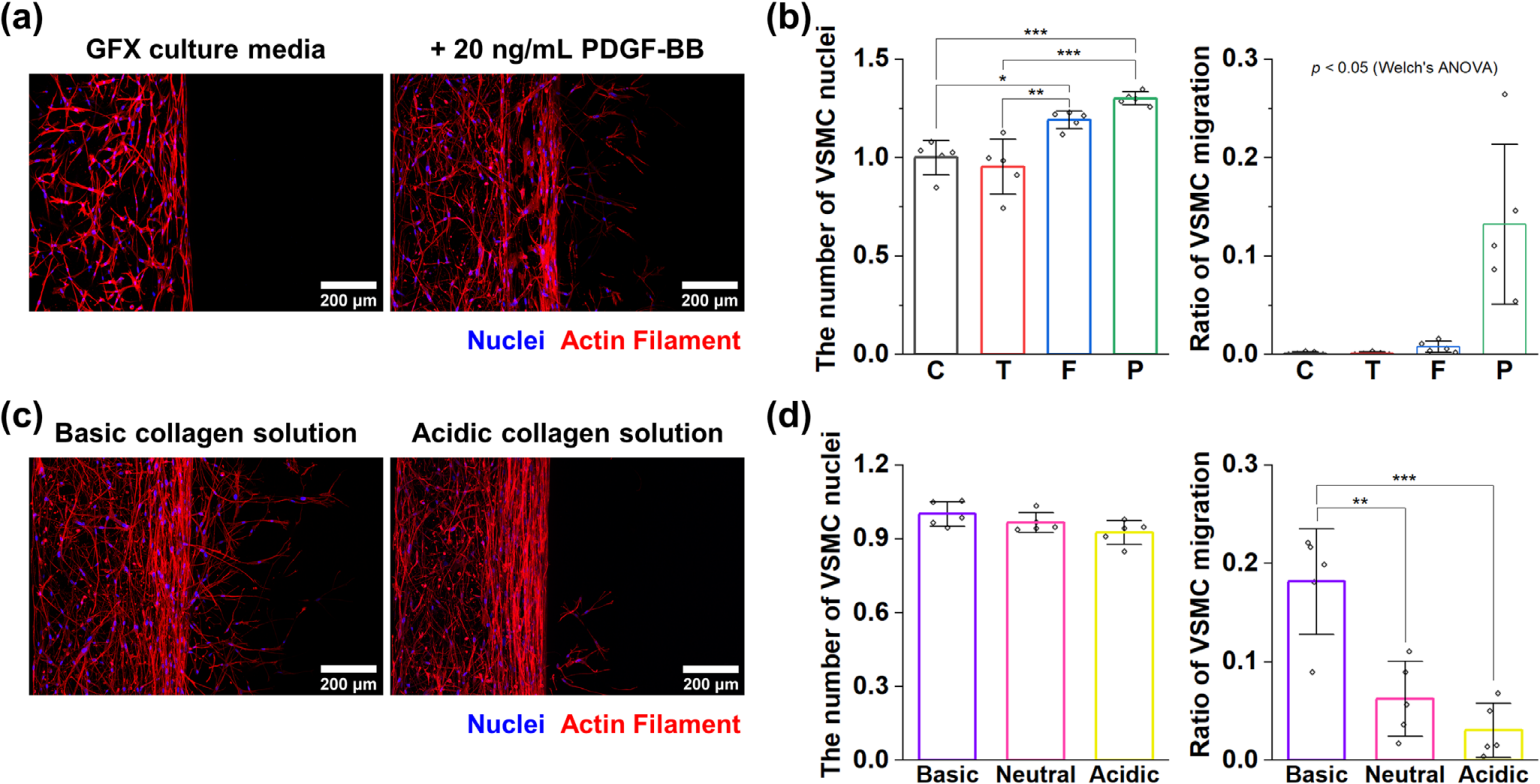
Investigation of optimal culture conditions for VSMCs in the intimal hyperplasia model. a) Representative collapsed confocal images of VSMCs according to the culture's medium conditions. b) Quantification of the number and migration ratios of VSMC (n = 5 devices). The number of VSMC nuclei was normalized to the average value for the C group. c) Representative collapsed confocal images of VSMCs according to the condition of the collagen in the subendothelial layers. d) Quantification of the number and migration ratio of VSMC according to the condition of collagen in the subendothelial layers (n = 5 devices). The number of VSMC nuclei was normalized to the average value for the basic group. Graphs represent the mean ± SD. Samples from at least two independent experiments were used for analysis. Significance was determined using one‐way ANOVA followed by Tukey's post‐hoc mean comparison between the two groups or using Welch's ANOVA followed by Dunnett T3 mean comparison between the two groups. GFX‐culture media: Growth Factor‐excluded culture media. C: Control (GFX‐culture media). T: + Thrombin 1U mL^−1^. F: + bFGF 20 ng mL^−1^. P: + PDGF‐BB 20 ng mL^−1^. *p < 0.05, **p < 0.01., ***p < 0.001.

To explore the conditions for proliferation and migration of VSMCs, which is one of the key characteristics of IH, we first investigated the effects of biomolecules on VSMCs. Thrombin, basic fibroblast growth factor (bFGF), and platelet‐derived growth factor (PDGF)‐BB, which are related to vascular injury and known to contribute to IH, were selected as the biomolecules for investigation.^[^ ^1^, ^27^, ^28^ ^]^ For this purpose, we used the 5‐channel MPS developed in our previous study (**Figure** **1**).^[^ ^17^ ^]^ In preparation of 3D VSMC culture, a basic collagen solution was introduced into the subendothelial channels, and a mixture of VSMCs and neutral collagen solution was seeded into the medial channels in the microphysiological device. In the culture of 3D VSMCs, growth factor‐excluded culture media (GFX‐CM) was used as a control, and GFX‐CM supplemented with 1 unit per mL (324 µg mL^−1^) thrombin, 20 ng mL^−1^ bFGF, or 20 ng mL^−1^ PDGF‐BB was used. In this experiment, thrombin had little effect on the proliferation and migration of VSMCs. In the presence of bFGF (**Figure** **2**a,b), proliferation of VSMCs showed a 1.19 ± 0.0460‐fold increase compared to the control group, and there was a slight increase in the migration ratio of VSMCs (0.00780 ± 0.00548) compared to the control group (0.00135 ± 0.00140). In the case of the addition of PDGF‐BB, there was a 1.30 ± 0.0331‐fold increase in VSMC proliferation compared with the control group, and the VSMC migration ratio (0.132 ± 0.0811) was also higher than the control group. Next, the properties of VSMC behavior were explored depending on the stiffness of the extracellular matrix. Based on a previous study, the stiffness of the collagen gel increases as the pH of the solution increases. Acidic (pH 6.7), neutral (pH 7.8), and basic (pH 9.9) collagen solutions were injected into the subendothelial layer, and then VSMCs were cultured using culture medium (CM) with 20 ng mL^−1^ PDGF‐BB.^[^ ^29^ ^]^ As the pH of the collagen solution decreased, the number of VSMCs slightly decreased, but there were no significant differences between conditions (Figure 2c,d). However, the migration of VSMC was 0.181 ± 0.0538 (basic collagen), which differed significantly from that of 0.0623 ± 0.0381 (neutral collagen) and 0.0304 ± 0.0275 (acidic collagen), confirming that the migration of VSMC decreased as the pH of the collagen solution decreased. Therefore, it was concluded that filling the subendothelial channel with a basic collagen solution and supplementing CM with PDGF‐BB in VSMC culture provides a suitable environment for pathological changes in VSMCs.

### 2.2 Establishment of Vascular‐Induced Intimal Hyperplasia Condition in Co‐Culture of ECs and VSMCs

**Figure 3:**
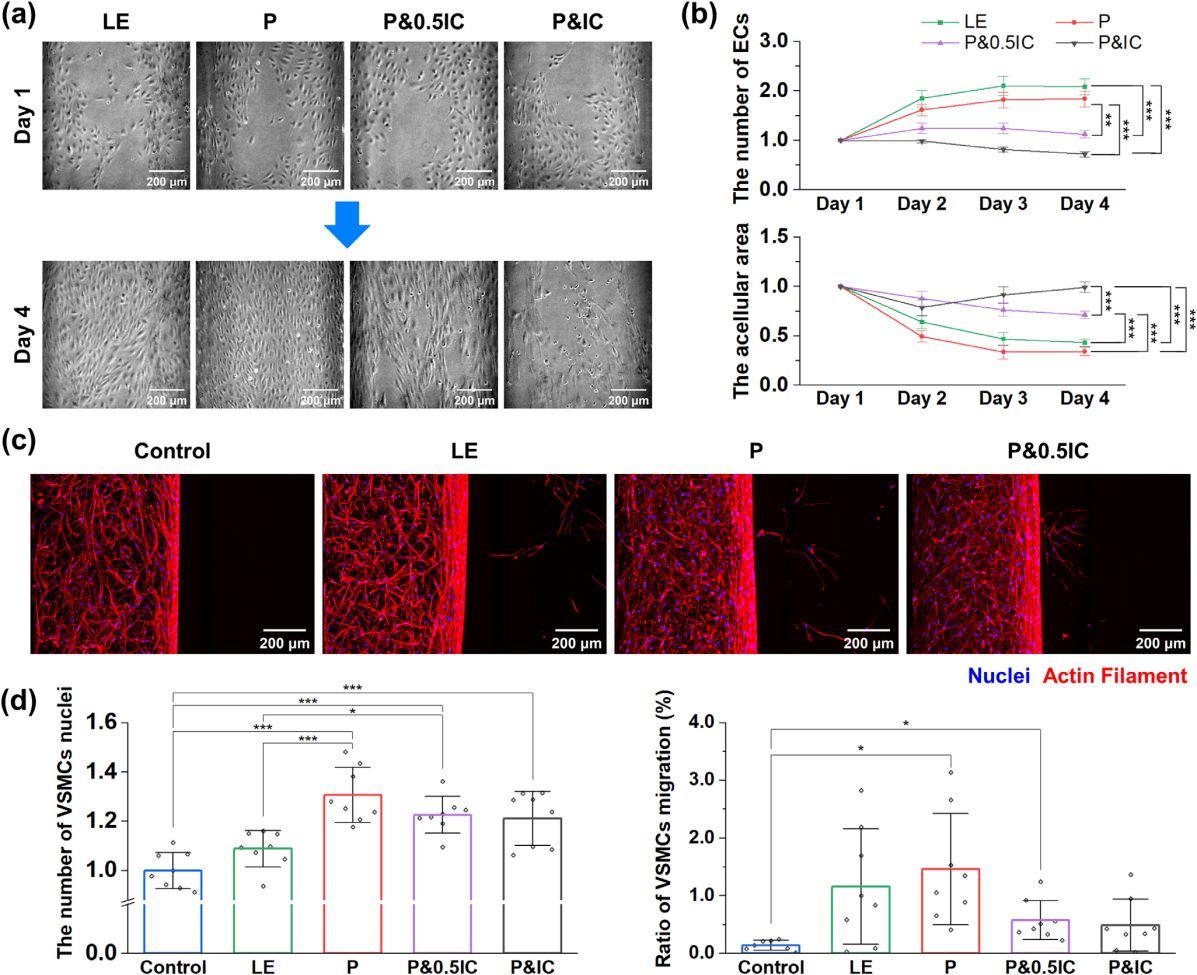
Establishment of the vascular injury‐induced intimal hyperplasia model. a) Representative phase‐contrast images of ECs according to the culture conditions. b) Quantification of the number of EC and acellular area in the endothelium according to the conditions (n = 8 devices). The values of days 2–4 for samples were normalized to the value of the corresponding day 1. Statistical analysis was conducted on day 4. c) Representative collapsed confocal images of VSMCs according to the culture conditions. d) Quantification of the number and migration ratio (n = 8 devices). The number of VSMC was normalized to the average number of VSMCs in the control group. Line graphs represent the mean ± SE. Bar graphs represent the mean ± SD. Samples from at least two independent experiments were used for analysis. Significance was determined using one‐way ANOVA followed by Tukey's post‐hoc mean comparison between the two groups or using Welch's ANOVA followed by Games‐Howell mean comparison between the two groups. The results of different statistical analysis are provided in Supporting Information. LE: Low‐concentration EC. P: LE+ 20 ng mL^−1^ PDGF‐BB. P & IC: LE + 20 ng mL^−1^ PDGF‐BB + inflammatory cytokine (1 ng mL^−1^ TNF‐α & 1 ng mL^−1^ IL‐1β). *p < 0.05, **p < 0.01., ***p < 0.001.

Among the pathological cascades of vascular injury‐induced IH, we decided to replicate the endothelial denudation, inflammation, and proliferation and migration of VSMCs. Inspired by previous research, we mimicked endothelial denudation by injecting a low concentration of ECs in the collagen‐coated EC channel of the device following the seeding of VSMCs (Figure 1b).^[^ ^30^ ^]^ To make inflammation and VSMC proliferation/migration in our disease model, we used PDGF‐BB and tumor necrosis factor (TNF)‐α and interleukin (IL)‐1β, which are involved in the vascular injury, in the 3‐day culture.^[^ ^31^ ^]^ Under the condition where a low concentration of HUVECs was seeded (LE condition) and cultured using CM for 3 days, the number of ECs increased, and the acellular area decreased rapidly (**Figure** **3**a,b). Similarly, when 20 ng mL^−1^ PDGF‐BB was added (P condition), the EC number increased and the denudation area decreased quickly. However, when 1 ng mL^−1^ of TNF‐α and IL‐1β was added along with PDGF‐BB (P&IC condition), the number of ECs gradually decreased, whereas the denudation area eventually increased. Because the damaged endothelium naturally recovers in the disease over weeks, the condition of exacerbating denudation was inappropriate as the disease model.^[^ ^31^ ^]^ Therefore, experiments were conducted under modified conditions where the concentrations of TNF‐α and IL‐1β were halved (P&0.5IC condition). Compared with the LE and P conditions, the P&0.5IC condition showed a gradual increase in EC numbers and a steady reduction in the denudation area. In the investigation of VSMC behavior, a high concentration of ECs seeded (control group) was added, and changes in VSMCs were observed across the conditions. Although the LE group showed a slightly higher number of VSMCs compared to the control group, the difference was not statistically significant (Figure 3c,d). However, significant differences were observed when PDGF‐BB was added to the medium. Specifically, the number of VSMCs increased by 1.31 ± 0.112‐fold in the P condition, 1.23 ± 0.0736‐fold in the P&0.5IC condition, and 1.21 ± 0.110‐fold in the P&IC condition compared with the control group. The migration ratio of VSMCs was significantly higher in the P (1.46 ± 0.966%) and P&0.5IC (0.574 ± 0.341%) conditions than in the control group (0.143 ± 0.0877%). Under the LE and P&IC conditions, the migration rates of VSMCs increased to 1.16 ± 1.00% and 0.491 ± 0.453%, respectively, although the difference was not statistically significant. Collectively, it was concluded that the P&0.5IC condition is appropriate for an in vitro disease model of vascular injury‐induced IH because the denudation of the endothelium could be maintained with a steady recovery, and the proliferation and migration of VSMCs were upregulated. Therefore, the P&0.5IC condition was designated as the IH condition.

### 2.3 Characterization of Endothelial Inflammation in the IH Model

**Figure 4:**
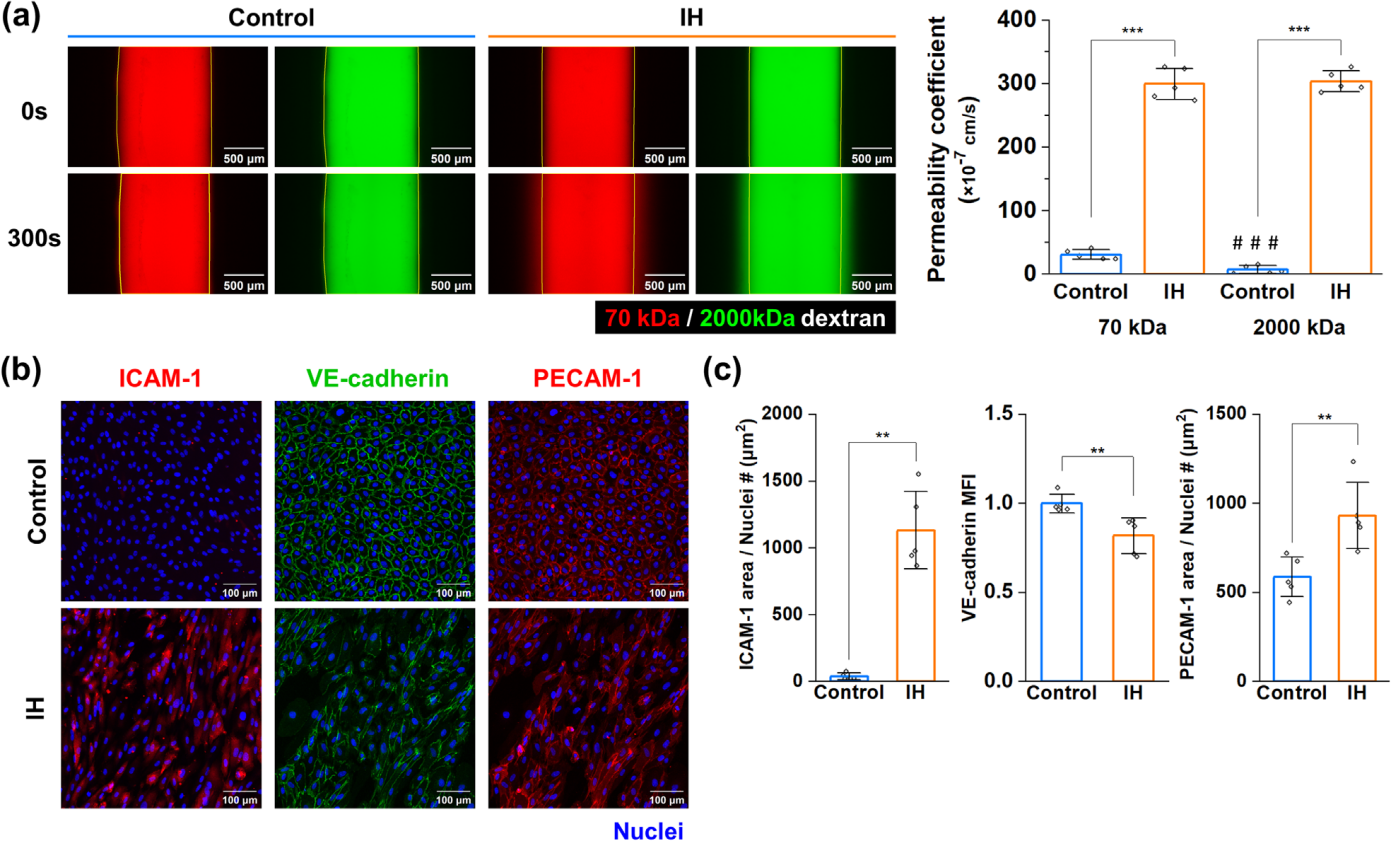
Characterization of the vascular injury‐induced intimal hyperplasia model. a) Fluorescent images of the permeability test (left) and quantification of the permeability coefficient for each condition (right, n = 5 devices). Yellow lines in the images represent the endothelial boundaries. # indicates statistical differences between dextrans under the same conditions. b) Representative projected confocal images of protein expression in endothelial cells. c) Quantification of protein expression (n = 5 devices). MFI of VE‐cadherin was normalized to the average value of the control group. Graphs represent the mean ± SD. Samples from at least two independent experiments were used for analysis. Significance was determined using an unpaired two‐tailed *t*‐test between two conditions. IH: intimal hyperplasia. MFI: Fluorescence intensity. *p < 0.05, **p < 0.01, ***p < 0.001, ^###^ *p <* 0.001.

The characteristics of endothelial inflammation in the IH model were investigated using fluorescence imaging and analysis. In the permeability assay, the IH model showed a significant increase in the permeability coefficients of both 70 and 2000 kDa dextran (2.99 ± 0.243 × 10^−5^ cm s^−1^ and 3.03 ± 0.166 × 10^−5^ cm s^−1^, respectively) compared to those of the control group (3.07 ± 0.753 × 10^−6^ cm s^−1^ and 7.02 ± 6.15 × 10^−7^ cm s^−1^, respectively) (**Figure** **4**a). Moreover, whereas the control group exhibited a significant difference in permeability coefficients between dextrans of different molecular weights, such a difference was not observed in the IH model. Next, the expression of proteins in endothelial cells was examined using immunofluorescence staining (Figure 4b,c). The expression of intracellular adhesion molecule‐1 (ICAM‐1), which is upregulated during inflammation and contributes to immune cell adhesion, in the IH condition showed a significant upregulation in aspect of its area per the number of nuclei (1132 ± 291 µm^2^) compared to the control group (37.9 ± 26.0 µm^2^) and an increase in mean fluorescence intensity (MFI) was observed (Figure 4b,c; Figure S1, Supporting Information).^[^ ^32^ ^]^ The MFI of VE‐cadherin, an indicator of vascular integrity as an endothelial junction protein, significantly decreased in the IH model by a 0.819 ± 0.0997‐fold compared to the control group.^[^ ^33^ ^]^ However, expression of platelet endothelial cell adhesion molecule (PECAM‐1), which is expressed at intercellular junctions on ECs and plays a role in the migration of immune cells into sites of inflammation, in the IH model showed a significant increase in its area per cell (587 ± 111 µm^2^ for the control group and 932 ± 186 µm^2^ for the IH condition).^[^ ^34^ ^]^ There was no significant difference in the MFI of PECAM‐1 (Figure S1, Supporting Information). PECAM‐1 was mostly localized to the boundary of ECs in the control group, whereas the protein in most ECs of the IH group was delocalized throughout the cell surfaces. Increased PECAM‐1 area in IH conditions stemmed from protein redistribution. Such redistribution of PECAM‐1 indicates a weakened vascular integrity.^[^ ^35^ ^]^ Collectively, endothelial inflammation was upregulated in our IH model.

### 2.4 Treatment of Therapeutic Agents on IH Model

**Figure 5:**
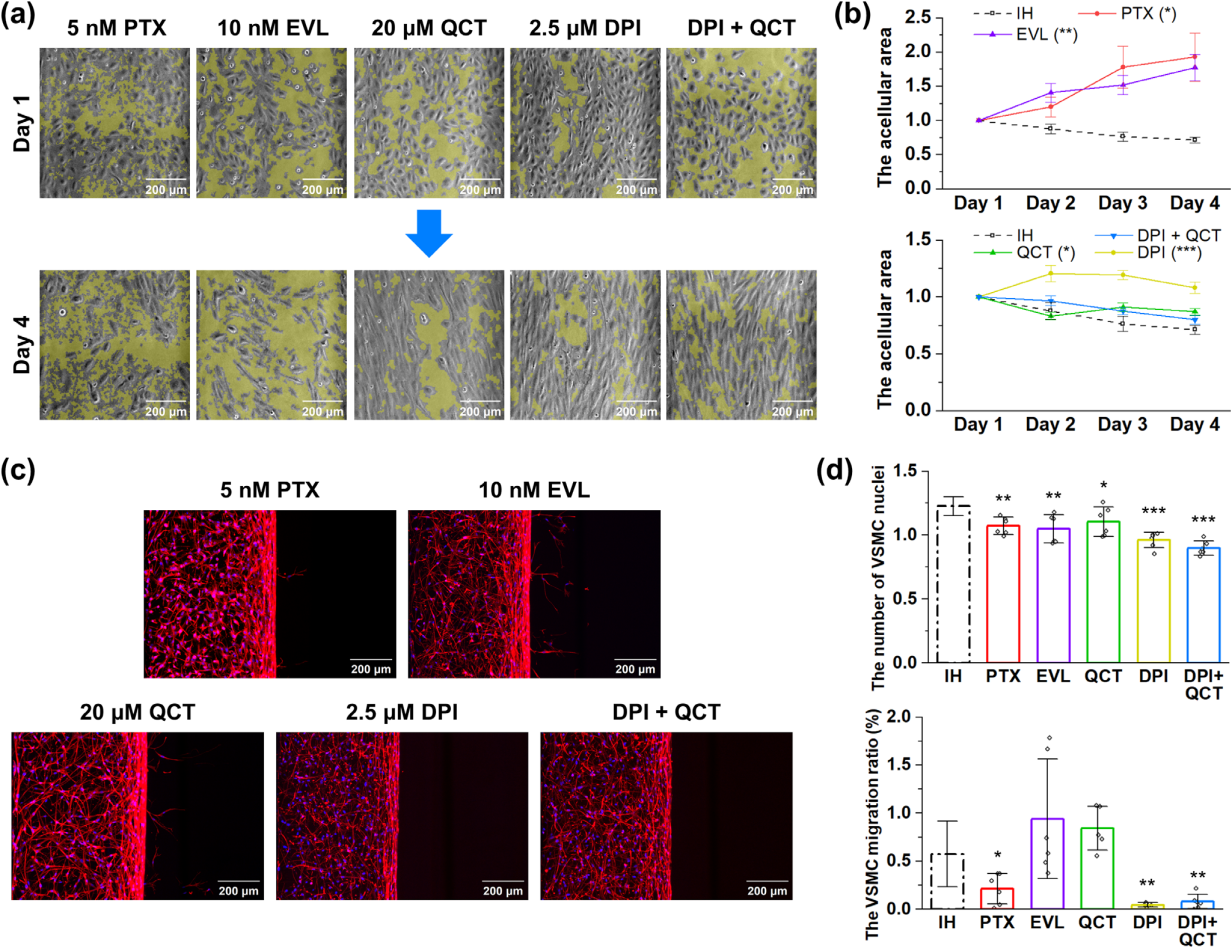
Investigation of IH model treated with therapeutic agents. a) Representative phase‐contrast images of ECs after drug supplementation. Yellow shades in the images indicate the acellular areas in the endothelium. b) Quantification of the acellular area in the endothelium according to conditions (n = 6‐8 devices). The values of days 2–4 for samples were normalized to the value of the corresponding day 1. Statistical analysis was conducted on day 4. c) Representative projected confocal images of VSMCs after drug supplementation. d) Quantification of the number and migration ratio (n = 6 devices). The number of VSMC was normalized to the average number of VSMCs in the control group. The dotted data points indicate the data shown in Figure 2. Line graphs represent the mean ± SE. Bar graphs represent the mean ± SD. Samples from at least two independent experiments were used for analysis. Significance was determined using an unpaired two‐tailed *t*‐test compared to the IH condition. The results of different statistical analysis are provided in Supporting Information. PTX: paclitaxel. EVL: everolimus. DPI: diphenyleneiodonium. QCT: quercetin. *p < 0.05, **p < 0.01., ***p < 0.001.

To verify our IH model as a potential drug‐testing platform, we treated the IH model with therapeutic agents. To evaluate the efficacy of drugs, we focused on attenuating the disease characteristics of our IH model: i) re‐endothelialization, ii) inhibition of VSMC proliferation and migration, and iii) mitigation of inflammation. Among them, re‐endothelialization and inhibition of changes in VSMCs were considered priorities in evaluating the effectiveness of drugs. This is because endothelial denudation induces the adhesion of platelets and immune cells, which initiates pathogenesis, and the proliferation and migration of VSMCs ultimately results in restenosis of the arteries. We first administered antiproliferative drugs to our IH model and evaluated their effects. Among the antiproliferative drugs, paclitaxel and everolimus were chosen because they have been used in DES and have different mechanisms of action: paclitaxel stabilizes microtubules, and everolimus inhibits mTOR.^[^ ^4^ ^]^ Because drugs coated on DCBs or DESs are released immediately after the surgery, they were administered simultaneously with the cytokines used in the IH condition. The concentration of these drugs was based on that used in previous studies.^[^ ^36^, ^37^ ^]^ In terms of re‐endothelialization, both paclitaxel and everolimus progressively exacerbated endothelial denudation, as evidenced by an increased acellular area observed on day 4, with significantly greater denudation compared to the IH condition (**Figure** **5**a,b). Increase of denudation with antiproliferative drugs was due to the apoptosis of endothelial cells (Figure S6, Supporting Information). Furthermore, the level of cellular oxidative stress significantly increased under treatment with antiproliferative drugs compared to the IH condition (Figure S7, Supporting Information). Compared to the IH model for VSMCs (Figure 5c,d), paclitaxel and everolimus significantly reduced the number of VSMCs (1.07 ± 0.0665 and 1.05 ± 0.112, respectively), whereas the migration of VSMCs was suppressed by paclitaxel alone (0.214 ± 0.159%).

When establishing the IH model, we observed that higher concentrations of inflammatory cytokines, indicating more severe inflammation, were associated with suppressed re‐endothelialization. Based on these findings, we focused on anti‐inflammatory drugs that have been examined in IH studies.^[^ ^1^ ^]^ Specifically, we used aspirin and dexamethasone, which reduce inflammation by inhibiting NF‐κB activation, as well as resveratrol and quercetin, polyphenolic compounds that activate SIRT1 and are reported to exert antioxidative and anti‐inflammatory effects.^[^ ^38^, ^39^ ^]^ In addition, we tested diphenyleneiodonium (DPI), which inhibits the production of reactive oxygen species (ROS) by targeting nicotinamide adenine dinucleotide phosphate oxidases (NOXs) in cells.^[^ ^40^ ^]^ The drug concentrations we used were within the range of concentrations reported in previous studies using 2D experimental models.^[^ ^41^, ^42^, ^43^, ^44^, ^45^ ^]^ First, aspirin was found to exacerbate denudation but suppress VSMC proliferation and migration compared to IH conditions (Figure S2, Supporting Information). Dexamethasone initially enhanced the recovery of denuded endothelium up to day 2 but showed no significant difference with the IH condition at the end. Although dexamethasone inhibited VSMC proliferation, it significantly upregulated VSMC migration. Resveratrol was effective only in suppressing VSMC proliferation, but not in VSMC migration, and negatively affected re‐endothelialization. Treatment with quercetin resulted in a decrease in the denuded area on day 4, but showed a slight yet statistically significant delay in endothelial recovery compared to the IH condition (Figure 5a,b). The apoptosis of endothelial cells in the quercetin condition was significantly lower than that observed with antiproliferative drugs (Figure S6, Supporting Information). Similar to the effect of resveratrol on VSMCs, quercetin effectively inhibitied VSMC proliferation (1.10 ± 0.115), whereas there was no significant and slight increase in VSMC migration (0.842 ± 0.228%) (Figure 5c,d). DPI exhibited detrimental effects on endothelial cells, followed by a tendency toward recovery, which resulted in a negative impact on re‐endothelialization, with a slightly widened denuded area in the endothelium on day 4 (Figure 5a,b). While the apoptosis of endothelial cells in the DPI condition was slightly higher than that of quercetin, it was still considerably lower than that observed with antiproliferative drugs (Figure S6, Supporting Information). Meanwhile, it inhibited both the proliferation and migration of VSMCs compared to IH (0.961 ± 0.0612 and 0.0461 ± 0.0216%, respectively).

**Figure 6:**
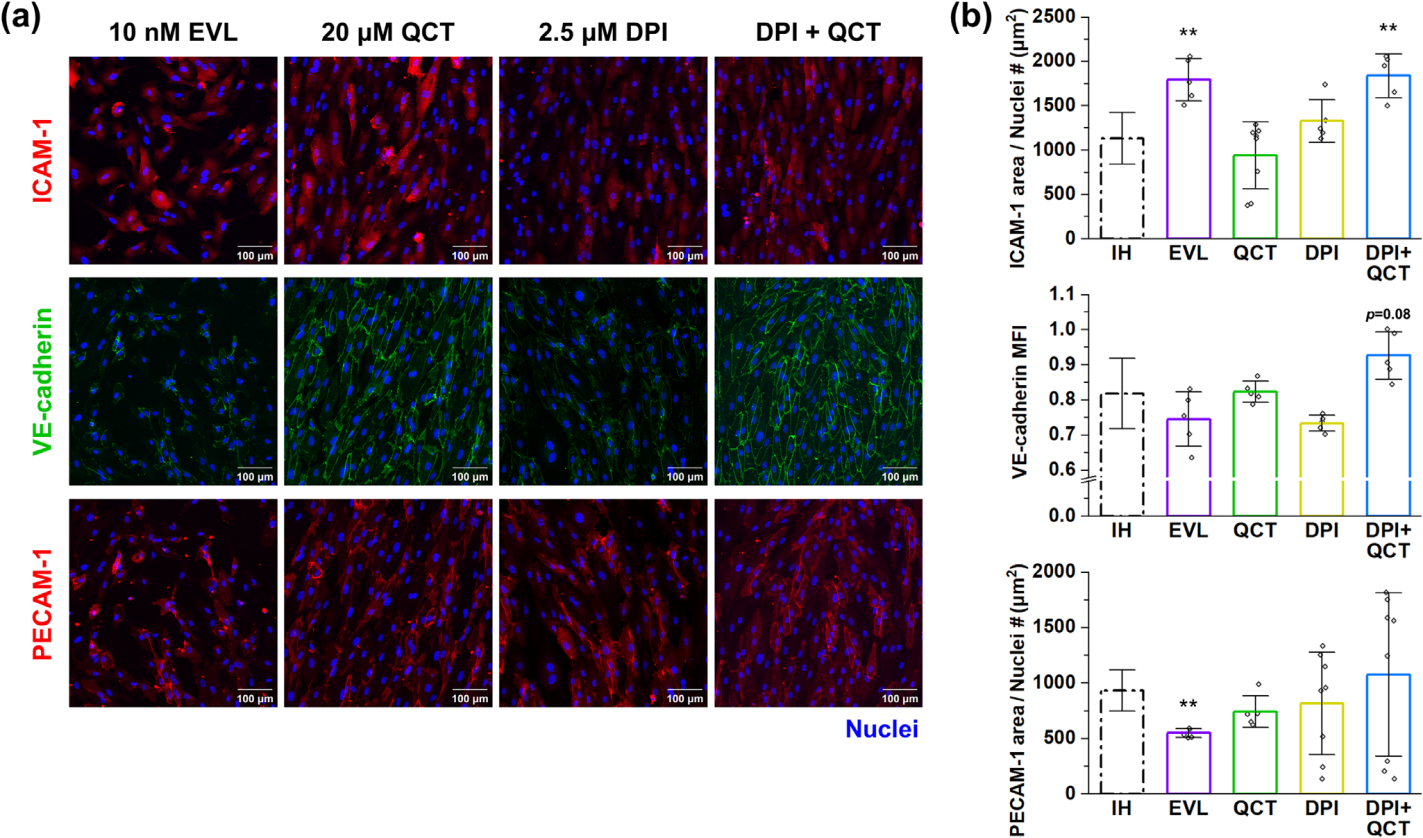
Investigation of protein expression in IH model treated with therapeutic agents. a) Representative projected confocal images of protein expression in endothelial cells. b) Quantification of protein expression (n = 5‐8 devices). MFI of VE‐cadherin was normalized to the average value of the control group. The dotted data points indicate the data shown in Figure 3. Graphs represent the mean ± SD. Samples from at least two independent experiments were used for analysis. Significance was determined using an unpaired two‐tailed *t*‐test or Mann‐Whitney U test between two conditions. The results of different statistical analysis are provided in Supporting Information. EVL: everolimus. DPI: diphenyleneiodonium. QCT: quercetin. MFI: Fluorescence intensity. **p < 0.01.

To investigate the changes in inflammation caused by drug treatment, we imaged and quantified the proteins tested in the previous section. Compared to the results under the IH condition (**Figure** **6**), everolimus treatment significantly increased ICAM‐1 expression (1795 ± 240 µm^2^) and significantly reduced PECAM‐1 expression (549 ± 40.7 µm^2^). VE‐cadherin expression was also reduced by everolimus (0.745 ± 0.0779), although the difference was not statistically significant. Aspirin showed no change in ICAM‐1 expression in endothelial cells, but a statistically significant decrease in VE‐cadherin and PECAM‐1 expression (Figure S3, Supporting Information). The reduction in PECAM‐1 under everolimus and aspirin conditions was primarily attributed to the presence of numerous cells with low protein expression, whereas PECAM‐1‐positive cells under both conditions exhibited delocalization. Compared to the IH condition, dexamethasone significantly increased PECAM‐1 expression, whereas the expression of other proteins showed no significant differences. In the case of resveratrol, the expression levels of all proteins showed no statistical differences compared to those in the IH condition. Similar to resveratrol, quercetin treatment had no significant effect on protein expression levels (Figure 6) compared to the IH condition (ICAM‐1: 942 ± 376 µm^2^, VE‐cadherin: 0.824 ± 0.0301, PECAM‐1: 742 ± 144 µm^2^). However, quercetin treatment resulted in a reduction in delocalized PECAM‐1. In the case of DPI treatment, compared with the IH condition, only slight differences were observed in the expression of ICAM‐1 expression (1330 ± 242 µm^2^), VE‐cadherin expression (0.734 ± 0.0229), and PECAM‐1 expression (816 ± 461 µm^2^).

### 2.5 Treatment of Combination of DPI and Quercetin on IH Model

In the experiments described in the previous section, quercetin has an effect on suppression of VSMC proliferation without adversely affecting re‐endothelialization, whereas DPI effectively suppressed VSMC proliferation and migration. In addition, neither drug significantly affected inflammation. Accordingly, we tried treating the IH model with these drugs, 2.5 µM DPI and 20 µM quercetin. With the previously denuded areas of the endothelium largely covered by ECs on day 4, the combined drug treatment exhibited a slightly slower recovery rate from endothelial denudation, but ultimately showed no statistically significant difference compared to the IH condition (Figure 5a,b). In particular, the apoptosis of ECs in the condition of drug combination was considerably lower than that in other drug conditions (Figure S6, Supporting Information). Furthermore, the combination significantly inhibited the proliferation and migration of VSMCs (0.898 ± 0.0552 and 0.080 ± 0.0757%, respectively) compared to the IH condition (Figure 5c,d). The inhibition of proliferation and migration by combining DPI and quercetin was confirmed using MTS and Transwell migration assays (Figure S8, Supporting Information). However, the expression levels of ICAM‐1 (1840 ± 248 µm^2^) significantly increased compared to the IH condition and the single treatment with either DPI or quercetin (Figure 6). In contrast, VE‐cadherin expression (0.926 ± 0.0674) showed a slight increase relative to the IH condition. According to PECAM‐1 expression of drug combination (1075 ± 735 µm^2^), there was no significant difference with the IH condition. Although the combination of DPI and quercetin showed marginal effects in inflammation according to the analysis of three related proteins, it was effective in the recovery from endothelial denudation and inhibition of VSMC proliferation and migration, which were of our primary importance in the evaluation of drugs. Thus, we investigated this drug combination, the control group, and the IH group.

### 2.6 Relative Secretomic Analysis Between 3D Artery Models

**Figure 7:**
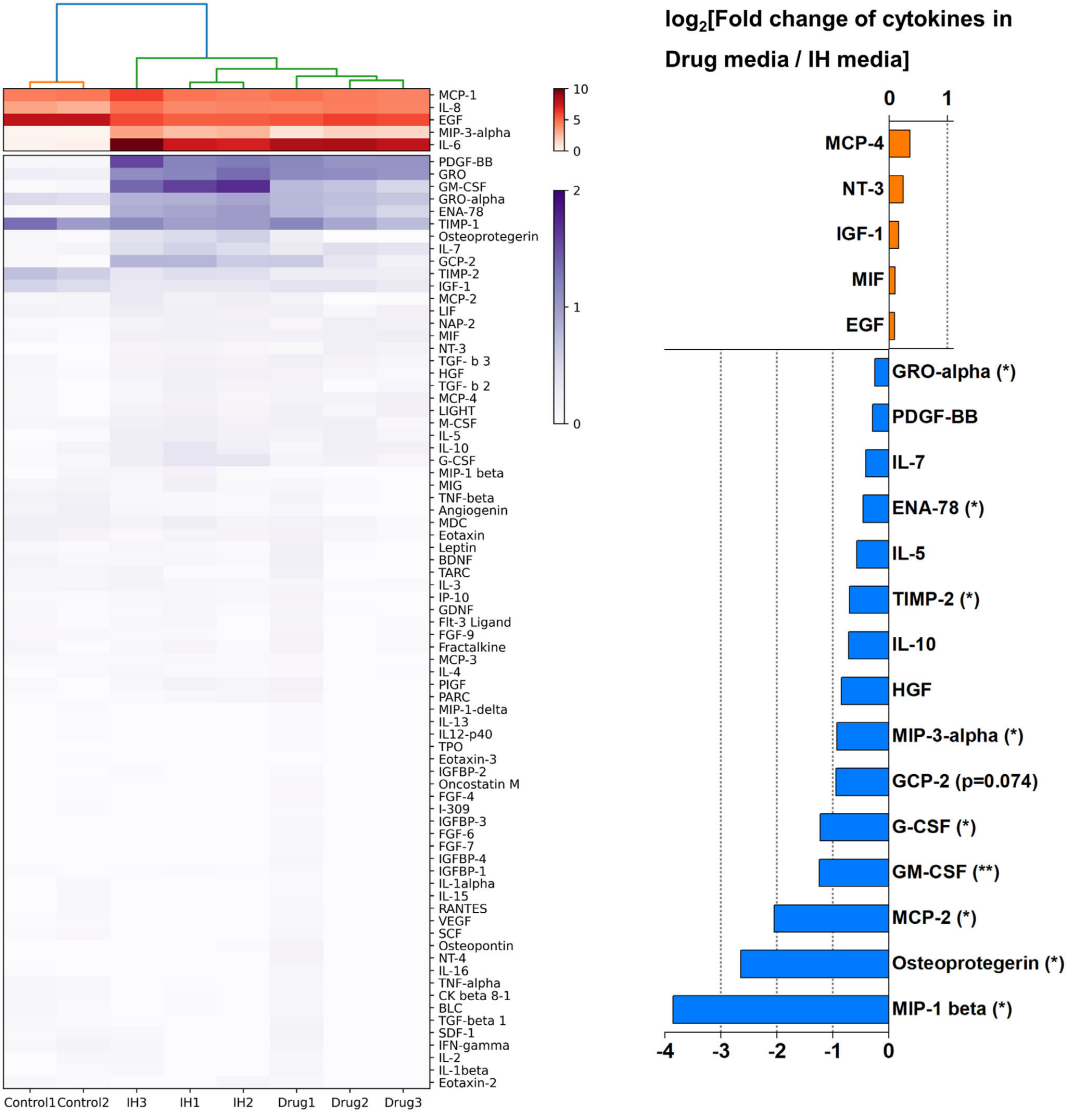
Investigation of cytokine secretion into the medium. Heatmap diagram of the relative amounts of 80 cytokines in the medium of each condition (left). The dendrogram above the heatmap diagram represents the hierarchical clustering of the samples. Hierarchical clustering of cytokines is shown in Supporting Information. Representation of log2‐fold mean difference in cytokines detected in medium from the IH condition over those from the IH with drug condition (right). Based on the p‐value from the an unpaired two‐tailed *t*‐test, the relatively high (top 5) and low (top 10) cytokines detected are represented (n = 3). Drug indicates 2.5 µM DPI and QCT (20 µM) were added to the IH medium. *p < 0.05, **p < 0.01.

To analyze the cytokines secreted under each condition, culture media were collected from days 2 to 4. Using the collected media, 80 cytokines were examined, and the results were expressed as a heatmap diagram with dendrograms (**Figure** **7**; Figure S4, Supporting Information). In addition, cytokines with relatively high levels (top 5) and low levels (top 10) between the IH condition and disease with drug condition are presented (Figure 7). MCP‐1 was secreted at similar levels across all conditions, whereas IL‐8, although the lowest in the control group, was also secreted abundantly under all conditions. EGF, IGF‐1, and TIMP‐2 were usually detected at higher levels in the control group than in the other groups. MIP‐3α, IL‐6, GRO, GM‐CSF, ENA‐78, and GCP‐2 were highly detected under IH and drug conditions compared with the control group. When comparing the IH condition to the drug‐treated condition, cytokines more abundantly secreted in the drug‐treated condition exhibited relatively small fold changes and no statistically significant change. However, cytokines secreted less in the drug‐treated group usually showed larger fold changes. Among these, an analysis of cytokines with statistically significant differences revealed a notable reduction in GRO‐α, ENA‐78, MIP‐3α, G‐CSF, GM‐CSF, MCP‐2, and MIP‐1β levels. In addition, osteoprotegerin was significantly downregulated in the drug condition compared to the IH condition. TIMP‐2, which was predominantly secreted in the control group, also showed a significantly reduced secretion level under the drug treatment condition compared with the disease condition. Because the results showing that the levels of certain inflammation‐related cytokines were reduced by drug treatment, while ICAM‐1 expression increased, appeared contradictory, we investigated immune cell adhesion under each condition. Immune cell adhesion was increased in both the IH and drug‐treated conditions compared to the control group (Figure S9, Supporting Information). Although there was a slight reduction in immune cell adhesion in the drug‐treated condition compared to the IH condition, the difference was not statistically significant. However, when normalized to 10000 µm^2^ of the EC area, immune cell adhesion was reduced in the drug‐treated condition relative to the IH condition. Since MMP2 and MMP9 are known to play critical roles in arterial remodeling, their concentrations under the conditions were evaluated using ELISA in both MPS and 2D culture systems (Figure S10, Supporting Information).^[^ ^46^ ^]^ As a result, MMP2 levels did not differ significantly across conditions. In contrast, MMP9 levels were significantly elevated under the IH condition compared to the control, irrespective of 2D or 3D system. Furthermore, treatment with the drug combination significantly reduced MMP9 levels compared to the IH condition. Notably, MMP9 secretion was significantly higher in the 3D system than in the 2D system. However, within both the 2D and 3D groups, significant differences were observed only between the control and IH conditions. The analysis of secreted proteins revealed that drug combination partially reduced inflammation and MMP9 secretion.

### 2.7 Characterization of Vascular Injury and Remodeling, Inflammation, and VSMC Phenotype‐Related Gene Expression

**Figure 8:**
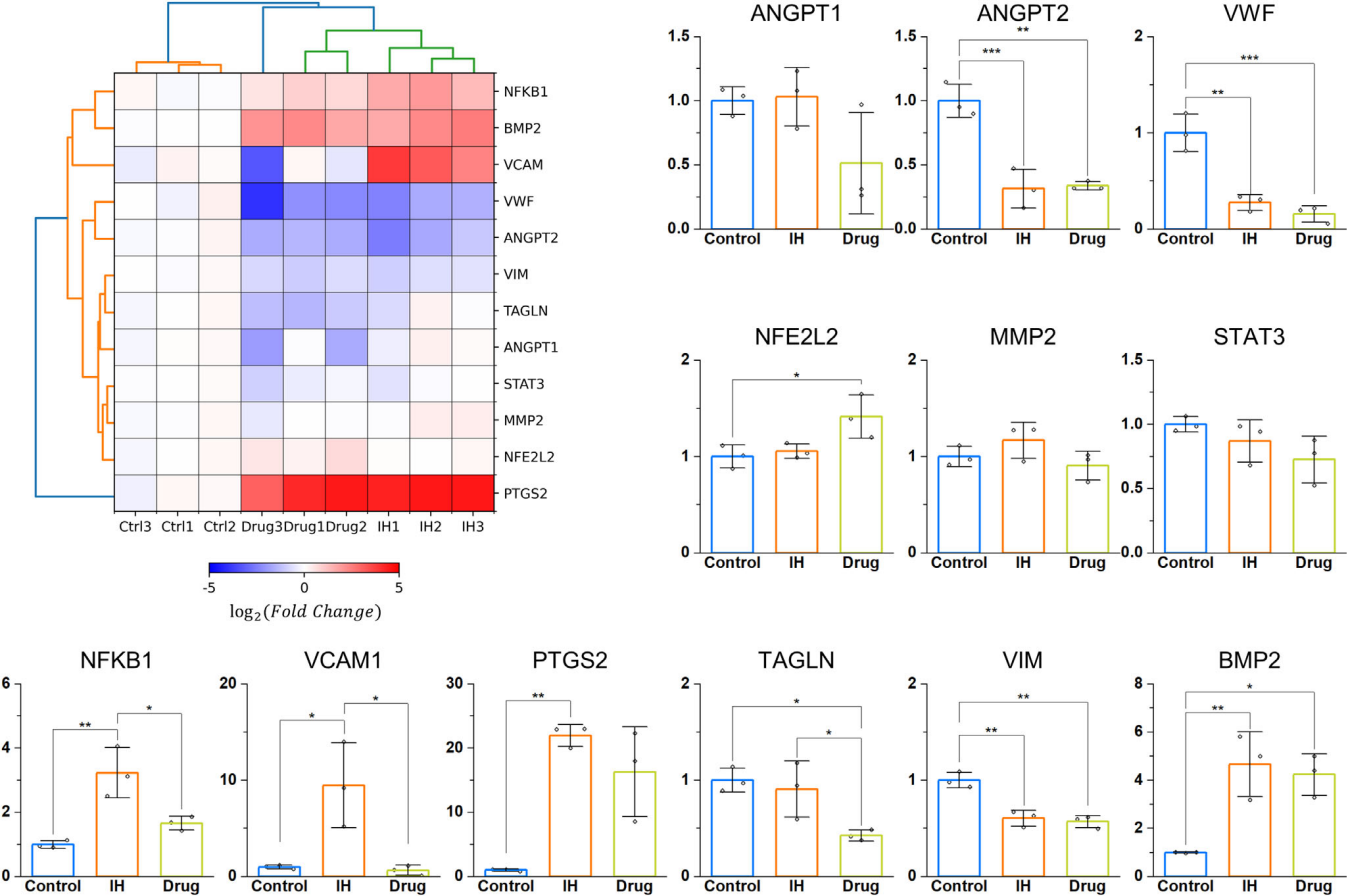
Quantification of gene expression related to vascular injury and remodeling, inflammation, and VSMC phenotype under various conditions. An overview of gene expression from RT‐qPCR is shown as a heatmap. Relative gene expression levels were quantified and are shown as bar graphs (n = 3). The gene expression levels were normalized to the average values of the control group. Bar graphs represent the mean ± SD. Significance was determined using one‐way ANOVA followed by Tukey's post‐hoc mean comparison between two groups or using Welch's ANOVA followed by Dunnett T3 mean comparison between the two groups. *p < 0.05, **p < 0.01., ***p < 0.001.

Based on previous studies, we investigated genes related to vascular injury and remodeling (ANGPT1, ANGPT2, VWF, NFE2L2, and MMP2), inflammation (STAT3, NFKB1, VCAM1, PTGS2, and CYBB), and the VSMC phenotype (TAGLN, VIM, BMP2, MYH11, and RUNX2) by isolating RNA from ECs and VSMCs together within the MPS device.^[^ ^47^, ^48^ ^]^ Among these genes, CYBB, MYH11, and RUNX2 were excluded from the analysis because their expressions were mostly undetermined. As shown in **Figure** **8**, ANGPT2 and VWF gene expression levels were significantly lower under IH (0.315 ± 0.152‐fold and 0.276 ± 0.809‐fold, respectively) and drug conditions (0.338 ± 0.0317‐fold and 0.159 ± 0.0861‐fold, respectively) than those in the control group. Von Willebrand factor (vWF) exhibited similar results in protein expression, as confirmed by immunofluorescence staining (Figure S5, Supporting Information). The expression level of the NFE2L2 gene was significantly higher in the drug group (1.414 ± 0.224‐fold) than in the control group. MMP expression was the highest under IH conditions, although there were no significant differences between the conditions. Except for the STAT3 gene, inflammation‐related genes were significantly upregulated under IH conditions. NFKB1 and VCAM1 exhibited significant reductions in expression under the drug treatment condition (1.66 ± 0.219‐fold and 0.644 ± 0.515‐fold, respectively) compared to the disease condition (3.23 ± 0.782‐fold and 9.47 ± 4.41‐fold, respectively). Compared to the control group, PTGS2 was upregulated under both IH and drug conditions (22.0 ± 1.70‐fold and 16.3 ± 7.00‐fold, respectively), with significant difference between control and IH conditions. The TAGLN gene showed the lowest expression under drug treatment conditions (0.427 ± 0.0572‐fold), with a significant difference compared to the other conditions (0.908 ± 0.294‐fold in the IH condition). The VIM gene exhibited the highest expression in the control group, showing a significant difference compared to the IH and drug conditions (0.605 ± 0.0825‐fold and 0.570 ± 0.0639‐fold, respectively). The BMP2 gene was significantly upregulated under IH and drug conditions (4.67 ± 1.35‐fold and 4.23 ± 0.872‐fold, respectively) compared to the control. As a result, we confirmed that drug treatment has a mitigating effect on the expression of inflammation‐related genes.

## 3 Discussion

To prevent restenosis after vascular intervention, DCBs and DESs have been used; however, adverse effects, including restenosis, have still been reported.^[^ ^2^, ^7^, ^8^, ^49^ ^]^ Although there is a need to explore new drug candidates to overcome the limitations of currently used antiproliferative drugs, existing experimental models also have shortcomings. Therefore, this study aimed to develop a suitable MPS‐based vascular injury‐induced IH model for testing therapeutic candidates. For this purpose, we first investigated biomolecules that induce pathological changes in VSMCs. The results showed that PDGF‐BB promoted VSMC proliferation and migration, bFGF upregulated VSMC proliferation, and thrombin had no effect compared to the control group (Figure 2a,b). The results regarding VSMC proliferation in response to biomolecules were consistent with those of previous studies, despite differences in the type of PDGF used.^[^ ^27^ ^]^ In addition, other studies have reported that PDGF and FGF promote VSMC proliferation and migration.^[^ ^50^, ^51^, ^52^ ^]^ However, the findings related to thrombin contradict those of earlier studies.^[^ ^53^, ^54^ ^]^ Thrombin affects VSMCs by inducing the production of growth factors, but the amount produced might be significantly lower than in other conditions, and the difference in results may be attributed to the 3D environment with firm collagen in our study compared to the 2D environment of a previous study.^[^ ^55^ ^]^ We also investigated the relationship between matrix stiffness and the behavior of VSMCs (Figure 2c,d). Previous studies have shown that arterial stiffness in disease conditions is higher than that in healthy arteries, and stiffer substrates cause the phenotypic transition of VSMCs from a contractile to a synthetic state.^[^ ^48^, ^56^ ^]^ Because we altered the stiffness of collagen exclusively in the subendothelial channel, the effects were localized to the cells near this channel. Because of this experimental setup, it is presumed that the overall number of VSMCs was minimally affected, but that the changes in the stiffness of subendothelial collagen significantly influenced cell migration.

Next, we established a vascular injury‐induced IH model with several characteristics of the disease, including endothelial denudation, VSMC proliferation and migration, and vascular inflammation, by introducing low concentrations of EC with a combination of PDGF‐BB, TNF‐α, and IL‐1β (Figure 3). To establish the disease model, we excluded detailed developmental processes of IH, such as platelet aggregation, fibrin layer formation, and immune cell involvement at the injury site.^[^ ^1^, ^5^ ^]^ Because these processes result in the production of various growth factors and inflammatory cytokines, we simplified them into exogenous growth factors and cytokines.^[^ ^1^, ^5^ ^]^ The growth factors were simplified to PDGF‐BB again because PDGF‐BB had the most significant impact on VSMC behavior in the aforementioned experiments. In addition, we simplified the inflammatory cytokines TNF‐α and IL‐1β, because TNF‐α and IL‐1β activate NF‐κB, which transcriptionally induces the production of inflammatory cytokines including IL‐6, IL‐8, and MCP‐1.^[^ ^38^, ^57^ ^]^ Therefore, secretion of various types of inflammatory cytokines was observed in the IH model (Figure 7). In patients with restenosis, plasma PDGF levels were reported to reach 2417 and 1367 pg mL^−1^ at 1 and 15 min after coronary angioplasty, respectively.^[^ ^58^ ^]^ These PDGF levels represent systemic concentration rather than local concentrations at the lesion site. Given that previous in vitro studies used 10–30 ng mL^−1^ PDGF to induce VSMC proliferation, the PDGF concentration used in our model is appropriate.^[^ ^27^, ^59^, ^60^ ^]^ Serum TNF‐α and IL‐1β levels in restenosis patients were approximately 40 and 150 pg mL^−1^, respectively, 24 h after stent implantation.^[^ ^61^, ^62^ ^]^ To mimic gradual recovery while maintaining endothelial denudation in the presence of PDGF in our model, we optimized TNF‐α and IL‐1β concentrations to 0.5 ng mL^−1^. Therefore, a potential improvement would be to adjust the ratio of TNF‐α to IL‐1β to better reflect clinical outcomes. Next, the established disease model showed increased endothelial permeability (Figure 4a). This was due to the loss of endothelial barrier function caused by denudation and inflammation.^[^ ^63^ ^]^ Because 2000 kDa dextran is close to the molecular weight of LDL, which has a molecular weight of 2.93 MDa, the increased permeability of this dextran suggests that LDL could accumulate below the endothelium of arteries after vascular injury.^[^ ^64^ ^]^ In addition, the established model exhibited characteristics of endothelial inflammation (Figure 4b,c). The upregulation of ICAM‐1 expression in ECs is commonly observed in inflamed blood vessels and has also been reported in the IH models in previous studies.^[^ ^65^, ^66^, ^67^ ^]^ Reportedly, blood vessels in inflammatory conditions downregulate VE‐cadherin expression in ECs, which is related to vascular permeability.^[^ ^63^, ^65^, ^68^ ^]^ It was also documented that PECAM‐1 on ECs is delocalized into the surface of the ECs in the presence of inflammatory cytokine.^[^ ^69^ ^]^

We also investigated the effect of antiproliferative drugs in our disease model (Figure 5). The finding that paclitaxel inhibits both the proliferation and migration of VSMCs, whereas everolimus suppresses proliferation but not migration in our study is consistent with previous studies.^[^ ^36^, ^70^, ^71^ ^]^ Although antiproliferative drugs delay the recovery of ECs, our study observed a further exacerbation of EC denudation.^[^ ^12^ ^]^ This is presumed to result from the augmentation of inflammation caused by the cytotoxic effects of the antiproliferative drugs.^[^ ^72^, ^73^ ^]^ Furthermore, increased ICAM‐1 expression and decreased VE‐cadherin expression in ECs treated with everolimus support exacerbated inflammation (Figure 6). The findings of exacerbated endothelial denudation due to antiproliferative drugs may explain the persistent incidence of adverse effects, including restenosis, in the use of DCBs and DESs, as well as the increased incidence of events reported in some studies, despite the use of DCBs and DESs.^[^ ^2^, ^8^, ^9^, ^74^, ^75^ ^]^ We explored potential alternatives to antiproliferative drugs and tried treating agents that could alleviate inflammation. Quercetin enhances wound healing and protects blood vessels from inflammation, which explains our findings of delayed yet evident endothelial recovery compared to IH in the presence of quercetin (Figure 5).^[^ ^76^ ^]^ Moreover, the significant reduction in the number of VSMCs under quercetin treatment compared to IH conditions aligns with a previous study.^[^ ^44^ ^]^ However, quercetin did not significantly affect VSMC migration and inflammation, which contradicts previous studies.^[^ ^44^, ^77^ ^]^ This discrepancy is presumed to result from the relatively low concentration of quercetin compared to the high pathogenic cytokines in the IH condition. Although not statistically significant, treatment with DPI slightly increased inflammation, which may have modestly impaired endothelial recovery. VSMC proliferation and migration could be promoted by NOX activation and the presence of ROS, which could explain the observed decrease of VSMC changes in DPI treatment.^[^ ^54^, ^78^, ^79^ ^]^

Finally, we tried treating a combination of DPI and quercetin, expecting to achieve an additive effect from the positive effects of both drugs. Quercetin, with its ability to mitigate the cytotoxic effects of several drugs, appears to attenuate the negative effects of DPI, resulting in re‐endothelialization levels comparable to those observed in the IH condition.^[^ ^80^, ^81^, ^82^ ^]^ In addition, the effect of the combined drug treatment on VSMC behavior is presumed to be because of the effects of DPI. However, the expression of proteins related to inflammation under combination treatment showed conflicting results. For the expression of VE‐cadherin, whereas no significant difference was observed compared to IH, the combination treatment yielded more favorable outcomes than individual drug treatments. However, the expression of ICAM‐1 in combined drug treatment was significantly higher than that observed under the IH condition and individual drug treatments. Collectively, the drug concentrations used in previous studies based on 2D in vitro monoculture systems appear to yield different results when applied to our 3D co‐culture model. However, our disease model also used relatively high cytokine levels, suggesting that re‐evaluation and optimization of drug concentrations are necessary.

In the secretome analysis (Figure 7), MCP‐1 and IL‐8, which were elevated under all conditions, were presumed to be associated with a lack of nitric oxide production due to the absence of shear stress.^[^ ^83^, ^84^, ^85^ ^]^ Notably, MCP‐1 in an arterial model with perfusion was measured to be relatively low even under disease conditions in our previous study.^[^ ^17^ ^]^ EGF was highest in the control group and decreased in the IH condition, but there was a tendency for a slight increase under drug conditions, although no statistical significance was observed. While a study suggested that EGF alleviates endothelial dysfunction, further systematic investigation is needed.^[^ ^86^ ^]^ IGF‐1 also showed a similar trend to EGF, but it has protective effects on cardiovascular diseases.^[^ ^87^ ^]^ Compared to the control group, the secretion of other cytokines was increased under disease conditions, and some of these cytokines showed a decrease under drug treatment conditions. GRO‐α, ENA‐78, MIP‐3α, G‐CSF, GM‐CSF, MCP‐2, and MIP‐1β levels were significantly reduced under drug conditions compared with disease conditions. These cytokines are associated with inflammation and the recruitment of immune cells (chemokines).^[^ ^88^ ^]^ The reduction in these cytokines suggests a decrease in inflammation and immune cell recruitment to the lesion, indicating the suppression of disease progression. In contrast, ICAM‐1 expression was higher than that observed under the IH condition (Figure 6), which appears contradictory to the reduced levels of proinflammatory cytokines. However, since ICAM‐1 is also implicated in endothelial repair, its sustained and elevated expression despite the decrease in proinflammatory cytokines under the drug treatment, where endothelial recovery was observed, may be attributed to this reparative function.^[^ ^89^ ^]^ Additionally, a reduction in immune cell adhesion was observed under the drug‐treated condition (Figure S9, Supporting Information). Therefore, further systematic investigations are needed to clarify the relationship between ICAM‐1 expression, chemokine secretion, and immune cell interactions. The increase in osteoprotegerin is strongly associated with intimal hyperplasia and restenosis, which is consistent with the elevated levels of osteoprotegerin observed in our disease model.^[^ ^90^ ^]^ The drug was effective in that the secretion of osteoprotegerin was significantly reduced to the level of the control group under drug condition. It is well known that MMPs and cathepsins play critical roles in arterial remodeling through their proteolytic functions, as well as in cellular processes such as migration, proliferation, and apoptosis via their non‐traditional proteolytic activities and involvement in intracellular signaling pathways.^[^ ^91^, ^92^, ^93^, ^94^, ^95^ ^]^ Specifically, MMP9 expression increases during the early stages of IH, whereas MMP2 expression becomes elevated as the disease progresses.^[^ ^46^ ^]^ In our study (Figure 8; Figure S10, Supporting Information), the disease condition was maintained for only three days; therefore, the observed upregulation of MMP9 secretion, along with unchanged MMP2 secretion and only a slight increase in MMP2 gene expression under IH conditions, are consistent with earlier findings. Furthermore, the treatment of drug combination could suppress arterial remodeling in the IH because MMP9 expression was reduced by drugs. In addition, MMP9 is presumed to be upregulated in the 3D culture within the MPS compared to the 2D culture on a well plate without collagen, due to its ability to degrade type I collagen.^[^ ^96^ ^]^ TIMPs are also closely associated with IH. While TIMP‐2 can inhibit MMP2 activity, it is also involved in the activation of pro‐MMP2.^[^ ^97^ ^]^ In our results, TIMP‐2 secretion was reduced under the IH condition compared to the control and further decreased under the drug treatment condition (Figure 7). Increased expression of ANGPT2, predominantly expressed in ECs, is associated with vascular remodeling and inflammation, and TAGLN and VIM are contractile and synthetic markers of the VSMC phenotype, respectively.^[^ ^98^, ^99^ ^]^ However, our results differ from those of previous studies (Figure 8). Control group's higher EC count possibly caused increased ANGPT2 expression. Because TAGLN and VIM are expressed in VSMCs as well as ECs, the results could be varied depending on the gene expression ratio of ECs and VSMCs.^[^ ^100^, ^101^ ^]^ Future investigations should consider analyzing gene expression exclusively in VSMCs to obtain features of their phenotype. The decrease in VWF expression promotes the proliferation and migration of endothelial cells, which is related to re‐endothelialization.^[^ ^47^ ^]^ In addition, upregulation of NFE2L2 in drug condition plays a role in cellular protection against oxidative stress and alleviates the transition of VSMC phenotype.^[^ ^47^, ^99^ ^]^ NFKB1, VCAM1, and PTGS2, which were upregulated in IH condition and diminished in drug condition, are related to vascular inflammation and arterial diseases.^[^ ^47^, ^102^, ^103^ ^]^ BMP2, which showed increased expression in the IH and drug conditions, is an osteogenesis marker of VSMCs.^[^ ^48^ ^]^

In this study, we recapitulated the acute stage of intimal hyperplasia induced by vascular injury using a microphysiological system. This stage is morphologically characterized by endothelial denudation, VSMC proliferation and migration, and inflammation.^[^ ^1^, ^31^ ^]^ In addition, we demonstrated intimal hyperplasia‐related pathological changes in the expression and secretion of relevant proteins, along with transcriptomic analysis of associated gene expression. Therefore, our 3D disease model closely resembles intimal hyperplasia both morphologically and physiologically and is reliable enough to reflect the actual disease. Furthermore, the drug combination we identified attenuated many of these pathological features, indicating it is at least effective within our model. However, several limitations remain. First, the thickness of the subendothelial layers and the proportion of VSMCs in our arterial model differ from those in actual arteries. In addition, the absence of mechanical stimuli may contribute to discrepancies between our model and the actual pathological environment. In particular, incorporating fluidic shear stress through perfusion, as well as hemodynamic alterations caused by stent implantation, could enhance the physiological relevance of the disease model. Furthermore, our model simplified several features of IH. Incorporating platelets and immune cells, especially monocytes and macrophages, in the lesion would improve its biological relevance. Extending the experimental duration would also be beneficial to capture later‐stage processes of clinical IH progression, such as ECM production and the sustained effects of IH‐targeting drugs. Lastly, it is necessary to validate the in vivo relevance and pharmacological findings presented in this study through comparative analyses with animal models in terms of pathological characteristics and drug responses.

## 4 Conclusion

In this study, we established a 3D model of vascular injury‐induced intimal hyperplasia using MPS. This model successfully mimicked endothelial denudation, proliferation, and migration of VSMCs, and inflammation of the disease. We found that antiproliferative drugs inhibited the proliferation of VSMCs, but aggravated endothelial denudation in our disease model. In addition, we showed partial improvements in DPI in VSMCs and quercetin in ECs in our model. Finally, we tried a combination of DPI and quercetin, which alleviated VSMC proliferation and migration and vascular inflammation without interrupting re‐endothelialization. This study yields a promising disease model and novel drug combinations, potentially transforming DCB and DES treatments.

## 5 Experimental Section

### Reagents and Cell Culture

Reagents used were listed as the name and its manufacturer: endothelial cell growth medium‐2 BulletKit (EGM‐2, Lonza); smooth muscle cell growth medium‐2 BulletKit (SmGM‐2, Lonza); 100× Antibiotic‐Antimycotic (Gibco); polydimethylsiloxane (PDMS, Dow); curing agent (Dow); dopamine hydrochloride (Sigma); 10 mm Tris‐HCl, pH8.5 (Biosesang); 10× phosphate‐buffered saline (Gibco); Phenol Red sodium salt (Sigma); Sodium hydroxide (Samchun); rat‐tail high concentration collagen I (Corning); endothelial cell basal medium‐2 (EBM‐2, Lonza); thrombin (Sigma); recombinant human PDGF‐BB(Peprotech); recombinant human bFGF(Peprotech); recombinant human TNF‐α(Peprotech); recombinant human IL‐1𝛽(Peprotech); dimethyl sulfoxide (DMSO, Sigma); paclitaxel (MedChemExpress); everolimus (MedChemExpress); dexamethasone (MedChemExpress); aspirin (MedChemExpress); resveratrol (MedChemExpress); quercetin (Sigma); diphenyleneiodonium chloride (Sigma); phosphate‐buffered saline (PBS, Gibco); TRITC dextran (Sigma); FITC dextran (Sigma); paraformaldehyde (PFA, Biosesang) solution; triton‐X 100 (Sigma); CAS block histochemical reagent (Invitrogen); bovine serum albumin (BSA, Sigma); Human Cytokine Array C5 (RayBiotech); TRIzol Reagent (Invitrogen); AccuPrep Universal RNA Extraction Kit (Bioneer); RNase‐Free‐DNase Set (Qiagen); AccuPower RocketscriptTM Cycle RT Premix (Bioneer); AccuPower 2X GreenStar Master Mix (Bioneer). The reagents used for the immunofluorescence staining are listed in Table S2 (Supporting Information).

Human umbilical vein endothelial cells (HUVEC, Angioproteomie) were cultured in EGM‐2 supplemented with 1% (v/v) 100× antibiotic‐antimycotic. Human aortic smooth muscle cells (HASMC, Lonza) were cultured with SmGM‐2 supplemented with 1% (v/v) 100× antibiotic‐antimycotic. HUVECs (passage 3–8) and HASMCs (passage 7–11) were cultured in an incubator (37 °C, 5% CO_2_) used for experiments.

### Formation of 3D Culture Models of Arterial Cells in MPS

The design and fabrication of a microphysiological device from our previous study was used.^[^ ^17^ ^]^ PDMS and the curing agent were mixed at an 8:1 (w:w) ratio, polymerized in a 55 °C oven for over 12 h, and then the polymerized PDMS was removed from the mold. The inlet and outlet of the EC channel were punched using a 6‐mm biopsy puncher and those of the other channels were punched using a 1‐mm biopsy puncher (Figure 1a). The sterilized PDMS and coverslip were bonded after O_2_ plasma treatment, then assembled devices were kept at 80 °C for at least overnight to restore hydrophobicity of their channels.

The experiment for 3D culture of arterial cells in MPS was conducted with slight modifications based on our previous study.^[^ ^17^ ^]^ The experimental procedures are shown in Figure 1b. Dopamine hydrochloride was dissolved in 10 mM Tris‐HCI at pH 8.5, and 3.0 mg mL^−1^ dopamine solution was injected into the subendothelial channels and ports of the medial channels and then treated for 2 h in an incubator (37 °C, 5% CO_2_). The solution was removed and the ports and channels of the device were washed twice with distilled water. Collagen solution (3.0 mg mL^−1^) was prepared using rat‐tail collagen type I solution, 10× PBS with phenol red, 0.05 N sodium hydroxide solution, and distilled water. The pH of the collagen solution was controlled by the ratio of the sodium hydroxide solution to distilled water, and the pH of the solution was measured using a digital pH meter (ISFETCOM). The subendothelial channels of the device were filled with a collagen solution, followed by incubation in an incubator (37 °C, 5% CO_2_) for over 30 min. Meanwhile, a cell mixture composed of 1.5 × 10^6^ VSMCs mL^−1^ in 3.0 mg mL^−1^ collagen solution (pH 7.0‐7.4). For the VSMC monoculture experiments, the EC channel of the devices was filled with 100 µL of EBM‐2 media, and the VSMC mixture was injected into the medial channels of the devices, followed by 30 min of incubation in an incubator (37 °C, 5% CO_2_).

For the EC‐VSMC co‐culture experiments, acellular collagen solution (0.05 mg mL^−1^) which 3.0 mg mL^−1^ collagen solution was diluted with the EBM‐2 solution, were additionally prepared. The EC channel of the devices was coated with 100 µL of 0.05 mg mL^−1^ collagen solution, and then the VSMC mixture was injected into the medial channels of the devices, followed by 30 min of incubation in an incubator (37 °C, 5% CO_2_). The coating solution in the EC channel was removed, and the channel was rinsed with 150 µL of EGM‐2 medium (CM). 150 µL cell mixture of 0.3 or 1.8 × 10^6^ HUVECs mL^−1^ in EGM‐2 medium was added into the EC channel. After incubation for 30 min (37 °C, 5% CO_2_), the unattached cells were removed and washed with 150 µL EGM‐2 solution.

### 3D Arterial Model Culture and Treatment of Drugs

A 24‐h incubation of samples with 300 µL GFX‐CM allowed investigation of biomolecule impact on VSMCs. For the next three days, the samples were cultured with 300 µL of GFX‐CM supplemented with 1 U mL^−1^ thrombin, 20 ng mL^−1^ bFGF, or 20 ng mL^−1^ PDGF‐BB. In the investigation of VSMC behavior based on the collagen stiffness of the subendothelial layer, the samples were cultured with 300 µL of CM for a day and cultured with 300 µL of 20 ng mL^−1^ PDGF‐BB‐added CM. In the EC and VSMC co‐culture experiments, the samples were cultured with 300 µL of CM on the first day of the experiment, followed by 300 µL of exogenous cytokine‐supplemented CM for the next 3 days.

Paclitaxel, everolimus, dexamethasone, resveratrol, quercetin, and DPI were dissolved in DMSO and mixed with CM at twice the intended concentration. Aspirin was directly dissolved in CM at twice the intended concentration owing to its solubility. The concentrations of drug solutions are presented in Table S1 (Supporting Information). Just before use, a twofold concentration of IH media (CM supplemented with 40 ng mL^−1^ PDGF‐BB, 1 ng mL^−1^ TNF‐α, and 1 ng mL^−1^ IL‐1𝛽) was mixed with the prepared drug solution at a 1:1 (v/v) ratio, resulting in the final medium containing the intended drug concentration along with 20 ng mL^−1^ PDGF‐BB and 0.5 ng mL^−1^ of TNF‐α and IL‐1𝛽. The drug‐containing medium was treated on the reconstructed arteries from day 1 to day 4. In all experiments, 300 µL of the medium was refreshed daily.

### Immunofluorescence Staining

The samples were washed twice with 200 µL of PBS for 5 min each, followed by fixation with 200 µL of PFA for 30 min. The fixed samples were washed four times with 200 µL of PBS for 5 min each, and then permeabilized of samples was conducted using 200 µL of 0.15% (v/v) Triton‐X 100 in PBS for 30 min, followed by PBS washing four times. The samples were blocked with 100 µL of the CAS block histochemical reagent for 15 min. The primary antibody was diluted with the blocking solution. After blocking, the solution with the primary antibody was added to the samples and incubated at 4 °C for 24 h. After incubation, the samples were washed four times with 0.1% (w/v) BSA in PBS for 15 min each. The secondary antibodies, DAPI, and phalloidin were diluted in 0.1% BSA‐containing PBS, which was loaded into the samples. After incubation at 4 °C for 24 h, the samples were washed four times again. The dilution ratios of the antibodies and probes are listed in Table S2 (Supporting Information).

### Image Acquisition by Microscopy

The confocal images in Figure 2 were acquired using LSM 880 (Carl Zeiss), and the confocal images in Figures 3, 4, 5, 6 were acquired using K1‐Fluo confocal microscopy (Nanoscope Systems). Confocal images were taken as a z‐stack with an interval of 4 µm for the 10× objective and 1 µm for the 20× objective. The region of interest (ROI) for the ECs was in the middle of the EC channel in the device, and the size of the ROI was 1257.8 µm × 1257.8 µm × 20 µm. ROIs for VSMCs were in the middle of both VSMC channels in the device, and their size was 1662 µm × 875 µm × 300 µm. Phase‐contrast images were obtained with an IX81 microscope (Olympus). Phase‐contrast images of ECs were taken every day from day 1 to day 4, and the size of the ROI was 1259 µm × 7000 µm.

### Image‐Based Analysis

Microscopic images were analyzed using FIJI ImageJ. Quantification of VSMCs was performed as follows: First, the total area of all slices corresponding to a single VSMC nucleus was measured. Distinguishable nuclei were selected under various conditions and the sum of the area for each of the 20 nuclei of the VSMCs was determined. The average VSMC nucleus area across slices was calculated to be 667.0 µm^2^. The number of VSMC nuclei in the ROIs was then determined by dividing the total nuclei area of VSMCs across all slices by 667 µm^2^. The ratio of migrated VSMCs was calculated as the total area of VSMC nuclei that had moved away from the main layer of VSMCs divided by the total nuclei area of VSMCs in the ROI.^[^ ^17^ ^]^ Phase‐contrast images of ECs were used to quantify the number of ECs and denuded areas of the endothelium. The number of ECs was determined within a central region of 500 µm × 6000 µm, and the “Enhance Local Contrast (CLAHE)” method was used to clearly distinguish the boundaries between ECs. The “analyze particles” method was then used to count only objects larger than 200 µm^2^. The acellular area of the endothelium was measured from a region of 800 µm × 800 µm using the PHANTAST plugin that was developed in a previous study.^[^ ^104^ ^]^ Confocal fluorescence images of ECs were used to quantify the MFI and the protein expression area per cell. MFI was calculated as the average fluorescence intensity detected in cells, excluding oversaturated fluorescence due to aggregation of the fluorescent probe and fluorescence detected in cell‐free areas. The protein expression area per cell was determined by dividing the total fluorescent area of the protein from ECs by the number of EC nuclei.

### Dextran Diffusion Assay

To assess permeability of the endothelium of the reconstructed arteries, a solution of 3.0 µm of 70 kDa dextran (TRITC) and 0.5 µm of 2000 kDa (FITC) was prepared. The medium of the reservoirs of the EC channel was aspirated, followed by 110 µL of dextran solution. Images were acquired from the central region of the device every 30 s for 5 min. The permeability coefficient (P) of the endothelium was derived from the following Equation (1).^[^ ^105^ ^]^

(1) $$P=\frac{1}{\Delta t}\;\frac{\left(I_{S,f}-I_{S,i}\right)}{\left(I_{E,i}-I_{S,i}\right)}\;\frac{V_{S}}{A_{E}}=\frac{1}{\Delta t}\;\frac{\left(I_{S,f}-I_{S,i}\right)}{\left(I_{E,i}-I_{S,i}\right)}\frac{A_{S}}{l_{E}}$$

In the formula, Δt is the experiment time between the first and last images, *I_E,i_* is the average fluorescent intensity of dextran in the lumen of the endothelium; *I_S,i_* and *I_S,f_* are the initial and final average fluorescent intensity in the subendothelial region, V_S_ and *A_S_* are the volume and area of the subendothelial region that receives dextran from the lumen, and *A_E_* and *l_E_* are the area and length of the endothelium region that the dextran traverses, respectively.

### Detection and Analysis of Cytokines

From days 2 to 4, 200 µL of the CM used on the chip was collected, and 600 µL of CM was collected per chip. Since a minimum of 1 mL was required for the assay, media collected from two chips were pooled to create samples. As a product manual, the collected media was centrifuged at 3000 × g for 10 min; media except for the pellet were stored at ‐80 °C. A cytokine array was conducted according to the manual provided by the manufacturer. Briefly, after blocking the membranes, 1 mL of sample (collected culture medium) was reacted with the membrane for 2 h. After washing, the membranes were treated with a biotinylated antibody cocktail for 2 h and washed. The membrane was incubated with HRP‐streptavidin for 2 h, washed, and then reacted with detection buffer for 2 min. Chemiluminescence of the membranes was acquired using a CCD camera (Cytiva) immediately after incubation. A series of experiments were conducted at room temperature. The chemiluminescent blot intensity on membranes was measured with ImageQuant TL v.10.2 (Cytiva). The intensity of spots lower than the mean intensity of the negative control spots (no antibodies) was considered, as the corresponding cytokine was not detected. Otherwise, the blot intensity was subtracted from the average intensity of the negative control spots, which was normalized to the average intensity of the positive control spots. Hierarchical clustering analysis between samples and between cytokines was conducted using the average linkage method. Assay data appear as a heatmap; hierarchical clustering, a dendrogram.

### Real‐Time Quantitative Polymerase Chain Reaction (qPCR) and Analysis

ECs and VSMCs within the device were lysed using TRIzol on day 4 of the experiment, and total RNA was extracted using an AccuPrep Universal RNA Extraction Kit according to the manufacturer's instructions. Genomic DNA (gDNA) was removed using an RNase‐Free DNase Set. The extracted RNA was then synthesized into complementary DNA (cDNA) using the AccuPower Rocketscript Cycle RT Premix on an AllInOneCycler PCR system (Bioneer). Next, real‐time qPCR arrays were conducted using an Exicycler 384 Real‐Time Quantitative Thermal Block (Bioneer). The reaction mixture used in the real‐time qPCR comprised 3 µL cDNA, 3 µL diethyl pyrocarbonate distilled water, 7.5 µL AccuPower 2X GreenStar Master Mix, and 1.5 µL primer set. Cells from the six devices were pooled into independent samples and used for real‐time qPCR analysis. We tested each gene three times. Genes with at least one undetermined expression reading were considered abnormal and removed. The expression levels of target genes were determined using the ΔΔC_T_ method and the expression level of the reference gene (GAPDH). Hierarchical clustering analysis between samples and between genes was conducted using the average linkage method. The data from the assay are plotted as bar graphs and a heatmap diagram, and their hierarchical clustering is expressed as a dendrogram. Primer sequences for the target genes are listed in Table S3 (Supporting Information).

### Data Representation and Statistics

Data were preprocessed as follows: the number of VSMC nuclei and the MFI of each protein were normalized using the respective average values of the control condition. The number of ECs and the acellular area from day 2 to day 4 under each condition were normalized to the average values from day 1 of the corresponding condition. Outliers were identified using two methods: the interquartile range (IQR) method and Grubbs' t‐test. Specifically, data points were considered outliers and excluded from the analysis if they fell outside 1.5 times the IQR and were simultaneously identified as outliers by Grubbs' t‐test. Graphs and statistical analyses were generated using Origin 2020 (OriginLab) and Prism 10 (GraphPad Software). Bar graphs represent the mean ± standard deviation, and each data point is expressed as symbols around the graph. Line graphs are presented as mean ± standard error without data points to improve readability. The heatmap diagram and dendrogram were acquired using Python 3.12, using SciPy and Matplotlib libraries. Sample sizes for each experiment were indicated in the captions of the corresponding figures. Prior to statistical analysis, the Shapiro–Wilk test was performed to assess the normality of data within each group. When three or more groups were compared, Levene's test was used to evaluate the homogeneity of variances. If the assumption of homogeneity was met, One‐way analysis of variance (ANOVA) was used to compare the three or more conditions, followed by Tukey's post‐hoc mean comparison. If the assumption was violated, Welch's ANOVA was applied, followed by either the Games–Howell test (for sample sizes ≥ 6) or Dunnett's T3 test (for sample sizes < 6) as post hoc test. When comparing two conditions, an unpaired two‐tailed Student's t‐test was used. The Mann‐Whitney U test was performed when at least one of the two groups did not meet the assumption of normality. A p‐value less than 0.05 from the statistical test was considered a statistically significant difference and indicated as *, **, and ***, which denote *p <* 0.05, *p <* 0.01, and *p <* 0.001, respectively. The absence of denotation indicates *p >* 0.05, and there was no statistical difference.

## Conflict of Interest

The authors declare no conflict of interest.

## Supporting information



Supporting Information

## Data Availability

The data that support the findings of this study are available from the corresponding author upon reasonable request.
