# Supplementary material for: Novel Drug‐Testing Platform for Vascular Injury‐induced Intimal Hyperplasia Using a Microphysiological System
Source: Adv Healthc Mater. 2025 Aug 11;14(31):e00602. doi: 10.1002/adhm.202500602 (PMC12683234; doi:10.1002/adhm.202500602)

Supporting Information

Novel Drug Testing Platform for Vascular Injury-induced Intimal Hyperplasia Using a Microphysiological System

Ungsig Nam, HyeMi Kim, Jeong Ah Kim, Ki-Hwan Nam, Kye-Sung Lee, Hwan Hur, Jessie S. Jeon^*^ and Ji Yong Bae^*^

**Supplemental materials and methods**

**Detection of dead cells**

Before the assay, phase-contrast images of the ECs in MPS were captured to observe floating dead-cells or cell debris. After washing with 100 μL of EBM-2 twice, the samples were treated with 500 nM of propidium iodide (Invitrogen) and Hoechst 33342 (Invitrogen) in EBM-2 medium for 30 min. The solution was removed and washed with 100 μL of EBM-2 twice. The samples were observed right after washing.

**Immune cell adhesion assay**

U937 cells were cultured with RPMI 1640 medium supplemented with 10% (v/v) heat-inactivated fetal bovine serum and 1% (v/v) 100× antibiotic-antimycotic. U937 cells were activated using 10 nM phorbol 12-myristate 13-acetate (PMA, Sigma) for 48 h. Afterward, the activated U937 cells were treated with 10 μM CellTracker green CMFDA disolved in RPMI 1640 medium without serum for 30 min. The cells were washed with culturing medium and PBS, detached, and spun down. The cells were resuspended as 5 × 10^5^ cells/mL using respective conditioned medium. 50 μL of cell mixture was injected into the endothelium in MPS and incubated for 30 min. The cell mixture was removed and rinsed with 200 μL of PBS twice to remove non-attached cells, and then fixed with 4% PFA for 30 min. The samples were washed four times using 200 μL of PBS again, and then stained with DAPI and phalloidin.

**Cellular oxidative stress detection**

CellROX Deep Red reagent (Invitrogen) was added to the culture medium of the IH and antiproliferative drug conditions at a final concentration of 5 μM. Hoechst 33342 was also added to the solution. At the end of the culture period (day 4), the medium in the MPS was removed and replaced with 160 μL of assay medium. After incubation for 30 minutes at 37 °C, the assay medium was removed, and the samples were washed three times with 160 μL of EBM-2. The samples were imaged immediately after washing using a confocal microscope.

**2D cell culture and assays**

For the MTS assay, 5,000 VSMCs were seeded in each well of a 96-well plate and cultured for 3 days in conditioned medium. Subsequently, 20 μL of MTS reagent was added to each well containing 200 μL of medium and incubated for 1.5 h at 37 °C. After brief shaking on an orbital shaker, the absorbance at 490 nm was measured using a plate reader. The final absorbance for each well was calculated by subtracting the absorbance of an acellular well filled with the medium of the corresponding condition. Absorbance values for each condition were then normalized to the average absorbance of the control group.

For the migration assay, Transwells with a diameter of 6.5 mm and a pore size of 8 μm were placed in a 24-well plate. Then, 100 μL of EBM-2 medium was added to the upper chamber of each Transwell, followed by 200 μL of a cell suspension containing 2.5×10^5^ VSMCs/mL in EBM-2 medium, and 750 μL of conditioned medium was added to the separate lower wells. The Transwells were then transferred into the wells containing the conditioned medium and incubated at 37 °C for 24 h. After incubation, VSMCs in the Transwells were washed with PBS, fixed with 4% paraformaldehyde (PFA), and incubated at room temperature for 20 min. The fixed cells were washed and permeabilized with 0.15% Triton X-100. Subsequently, the cells were washed again and stained with DAPI. After staining, the Transwells were washed with PBS. Non-migrated VSMCs on the upper surface of the membrane were removed using a cotton swab. Migrated VSMCs on the lower surface were imaged by fluorescence microscope, and the number of nuclei was counted using FIJI. Hematoxylin and Eosin (H&E) staining was performed using an H&E Stain Kit (Vector Laboratories) according to the manufacturer's instructions.

**MMP2 and MMP9 secretion enzyme-linked immunosorbent assay (ELISA)**

To analyze the secretion of MMP2 and MMP9, the medium used in the MPS was collected and stored as described in Section 5.8 of the main text. To collect medium from 2D cultures, 36,000 VSMCs–the same number as used in the MPS model–were seeded in each well of a 48-well plate. Then, 300 μL of conditioned medium was used for cell culture and replaced daily. Approximately 200 μL of medium per well was collected over three days of culture and immediately stored at -80°C. Immediately before the assay, the frozen culture medium was thawed and centrifuged at 3000 × g to remove dead cells and cellular debris. MMP2 (Abcam) and MMP9 (Invitrogen) ELISAs were performed according to the manufacturers’ instructions. Briefly, 100 μL of medium from each condition was added in duplicate to microplates pre-coated with antibodies specific to the target proteins. Standard solutions and assay buffer (without protein) were also added to micro-wells in duplicate. The plates were incubated overnight at 4°C with gentle shaking. After washing, biotin-conjugated antibodies were added and incubated at room temperature for 1 hour with gentle shaking. Following another wash, HRP-streptavidin solution was added to the wells and incubated at room temperature with gentle shaking. After a final wash, TMB substrate solution was added and incubated with gentle shaking, followed by the addition of stop solution to terminate the reaction. Absorbance at 450 nm was immediately measured using a plate reader. All incubation times and reagent volumes were followed according to the manufacturers' protocols. MMP concentrations under each condition were estimated based on the standard curve. Absorbance values below that of the assay buffer were interpreted as zero concentration.


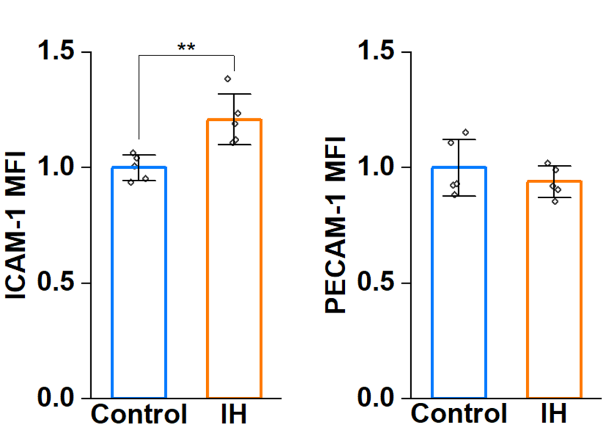


**Figure S1.** Mean fluorescence intensity of ICAM-1 and PECAM-1 expressed by ECs under control and IH conditions. MFI of each protein was normalized to the average value of the control group. Graphs represent the mean ± SD. Significance was determined using an unpaired two-tailed t-test between two conditions. ***p <* 0.01.


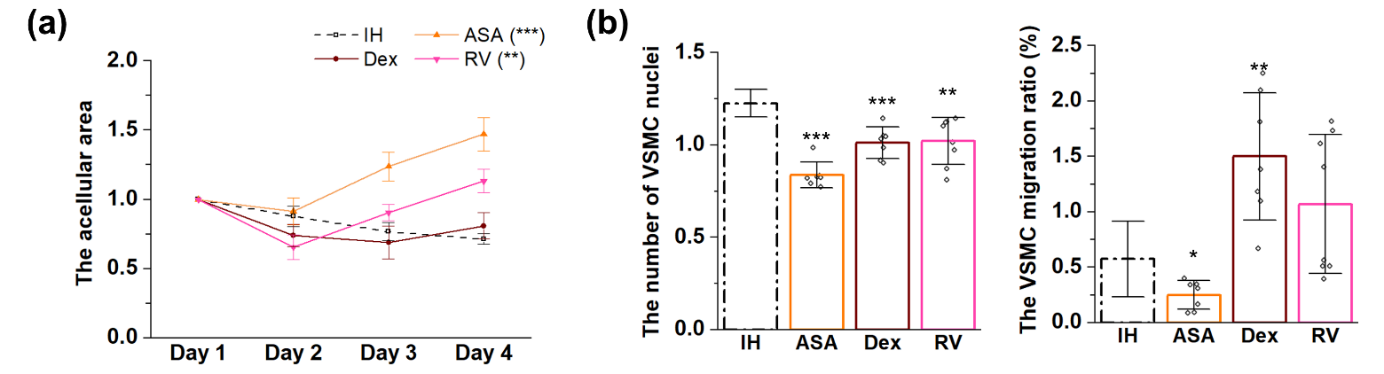


**Figure S2.** Additional investigation of the IH model treated with therapeutic agents. (a) Quantification of the acellular area in the endothelium according to conditions (n=8-9 devices). The values of days 2–4 ​​for samples were normalized to the value of the corresponding day 1. (b) Quantification of the number and migration ratio (n=6-7 devices). The number of VSMC nuclei was normalized to the average value of the control group. The dotted data indicate the data from Figure 2. (c) Line graphs represent the mean ± SE. Bar graphs represent the mean ± SD. Samples from at least two independent experiments were used for analysis. Significance was determined using an unpaired two-tailed t-test in comparison to the IH condition. ASA: acetylsalicylic acid (aspirin). Dex: dexamethasone. RV: resveratrol. **p <* 0.05, ***p <* 0.01., ****p <* 0.001.


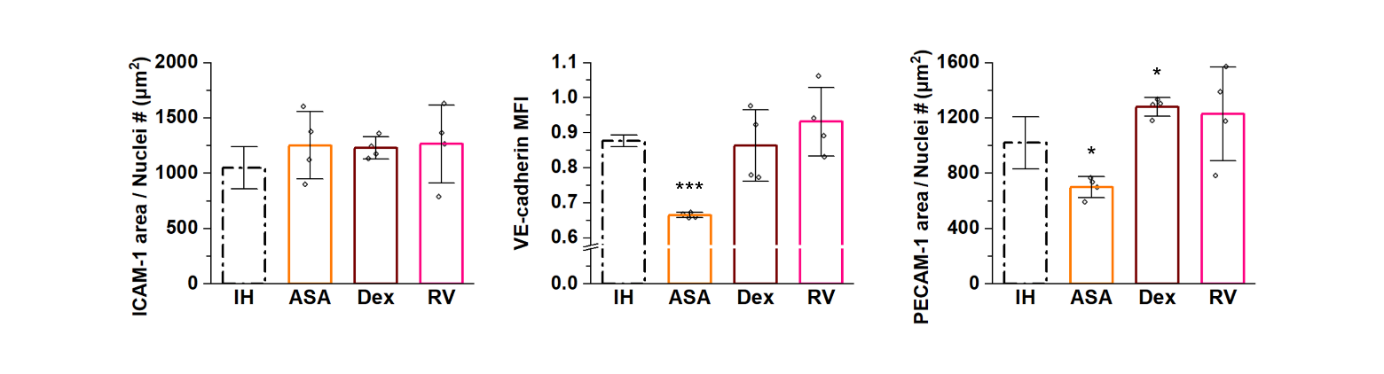


**Figure S3.** Quantification data for protein expression (n=3-4 devices). MFI of VE-cadherin was normalized to the average value of the control group. The dotted data indicate the data from Figure 3. Bar graphs represent the mean ± SD. Significance was determined using an unpaired two-tailed t-test in comparison to the IH condition. **p <* 0.05, ****p <* 0.001.


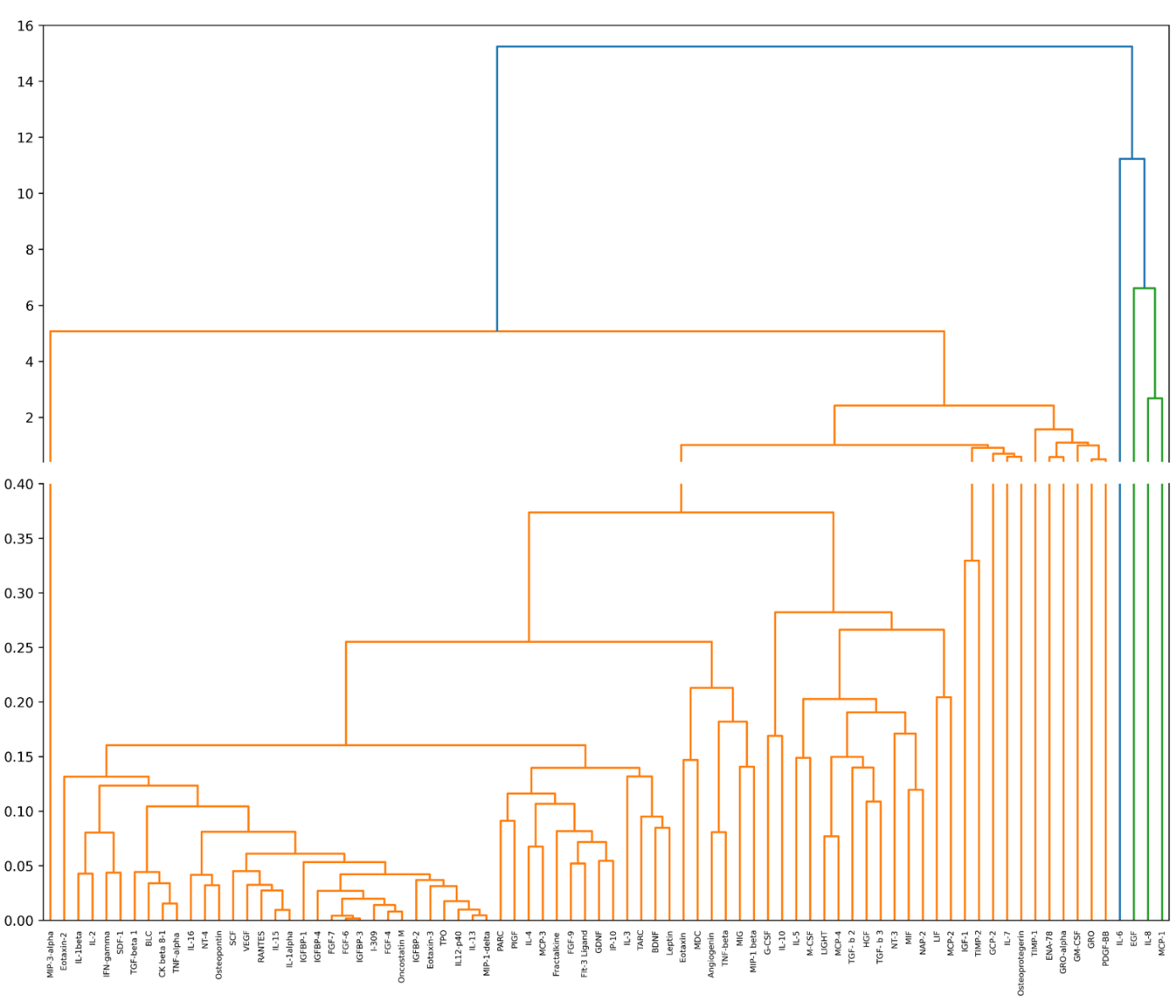


**Figure S4.** Dendrogram of hierarchical clustering of the 80 cytokines.


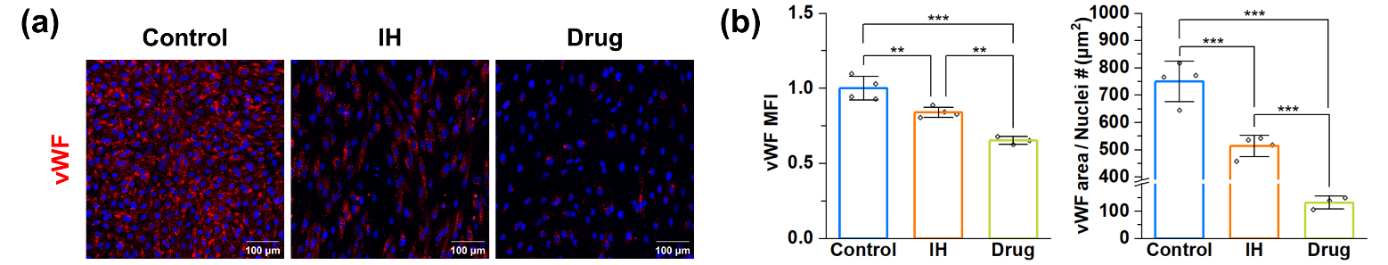


**Figure S5.** (a) Representative projected confocal images of von Willebrand factor (vWF) expression in endothelial cells. (b) Quantification of vWF expression (n=3-4 devices). MFI of vWF was normalized to the average value of the control group. Graphs represent the mean ± SD. Significance was determined using one-way ANOVA followed by Bonferroni’s post-hoc mean comparison between the two groups. **p <* 0.05, ***p <* 0.01., ****p <* 0.001.


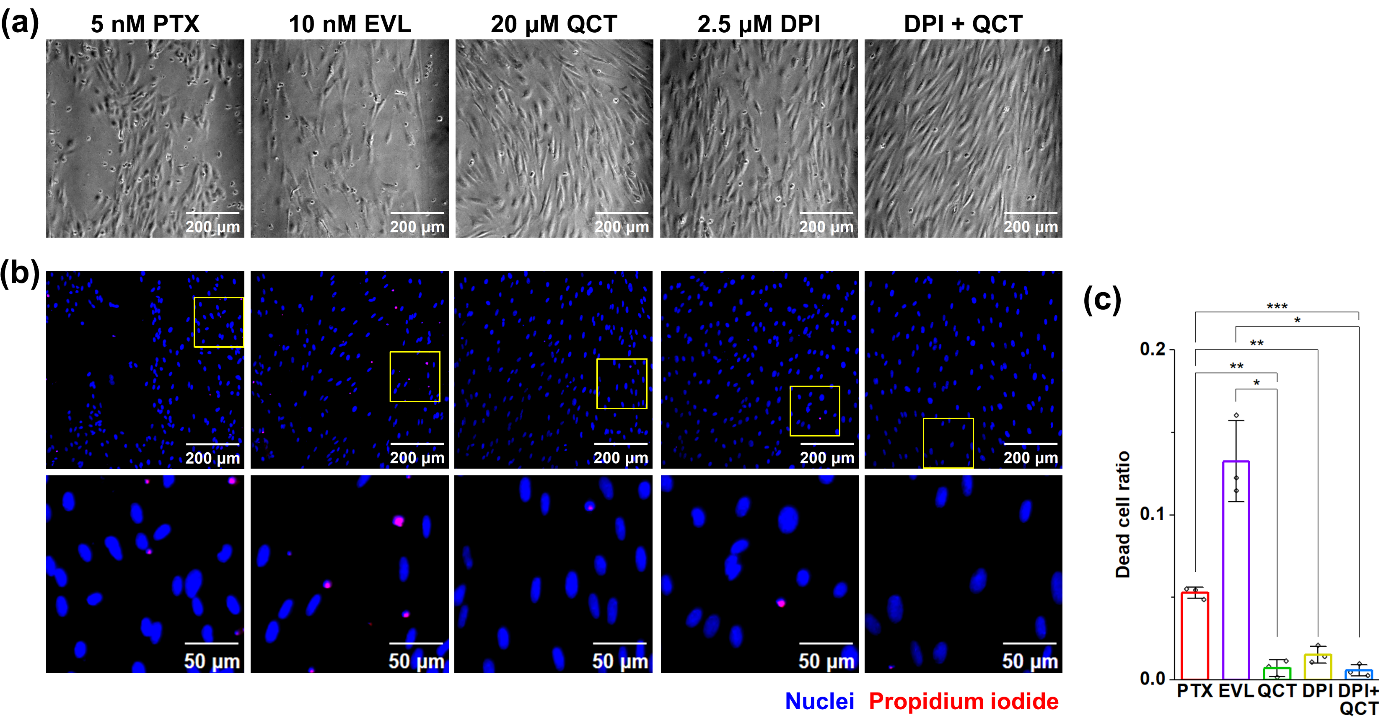


**Figure S6.** (a) Representative phase-contrast images of ECs before washing. Floating dead-cells or cell debris could be observed. (b) Fluorescent images of dead endothelial cells labelled with propidium iodide (top). Yellow box images were magnified (bottom). (c) Quantification of dead cell ratio (n=3 devices). Graphs represent the mean ± SD. Significance was determined using Welch’s ANOVA followed by Dunnett T3 post-hoc mean comparison between the two groups. **p < 0.05*, ***p < 0.01*., ****p < 0.001.*


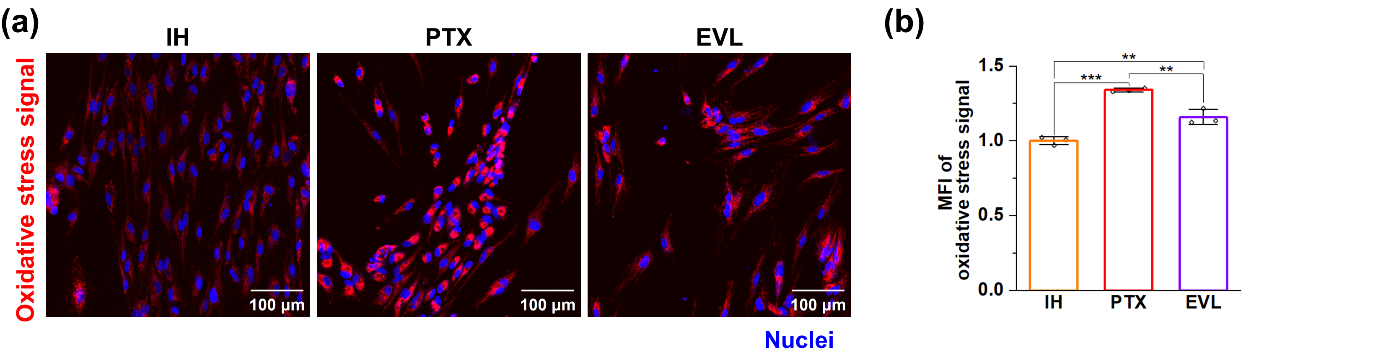


**Figure S7.** (a) Representative projected confocal images of oxidative stress signal of endothelial cells in each condition. (b) Quantification of oxidative stress of endothelial cells in each condition. The MFI was normalized using the average value of the IH group. n=3 devices. Graphs represent the mean ± SD. Significance was determined using one-way ANOVA followed by Tukey’s post-hoc mean comparison between the two groups. ***p < 0.01*., ****p < 0.001*.


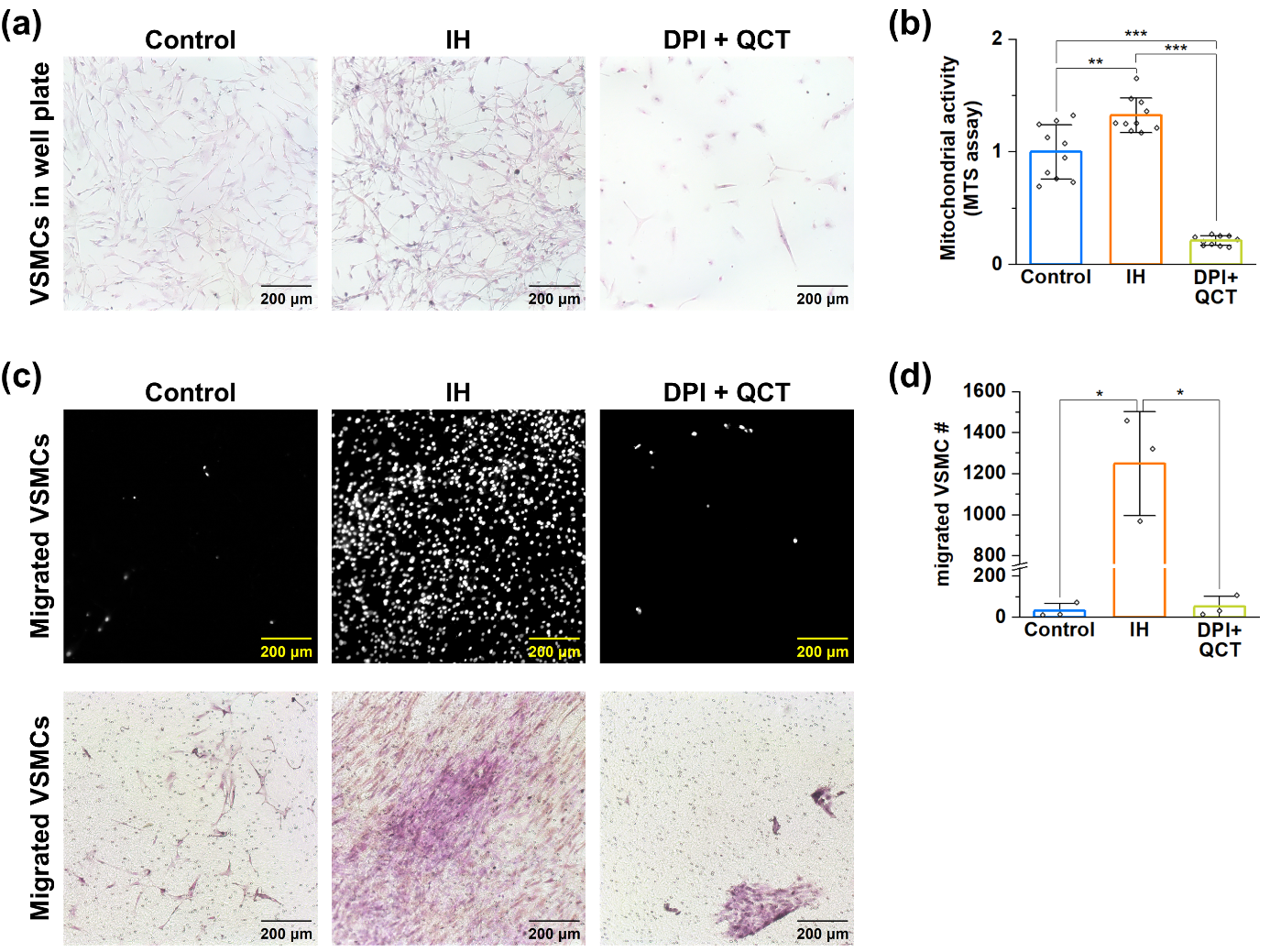


**Figure S8.** (a) Representative bright-field images of H&E-stained VSMCs in well-plate. (b) Quantification of mitochodrial activity of VSMCs, assessed by the MTS assay. (c) Representative fluorescent images migrated VSMCs in the Transwell (top) and bright-field images of H&E-stained migrated VSMCs in the Transwell (bottom). (d) Quantification of the number of migrated VSMCs in the Transwell assay. n=3. Graphs represent the mean ± SD. Significance was determined using Welch’s ANOVA followed by Dunnett T3 post-hoc in (b), and Games-Howell in (c) post-hoc mean comparison between the two groups. **p < 0.05*, ***p < 0.01*., ****p < 0.001.*


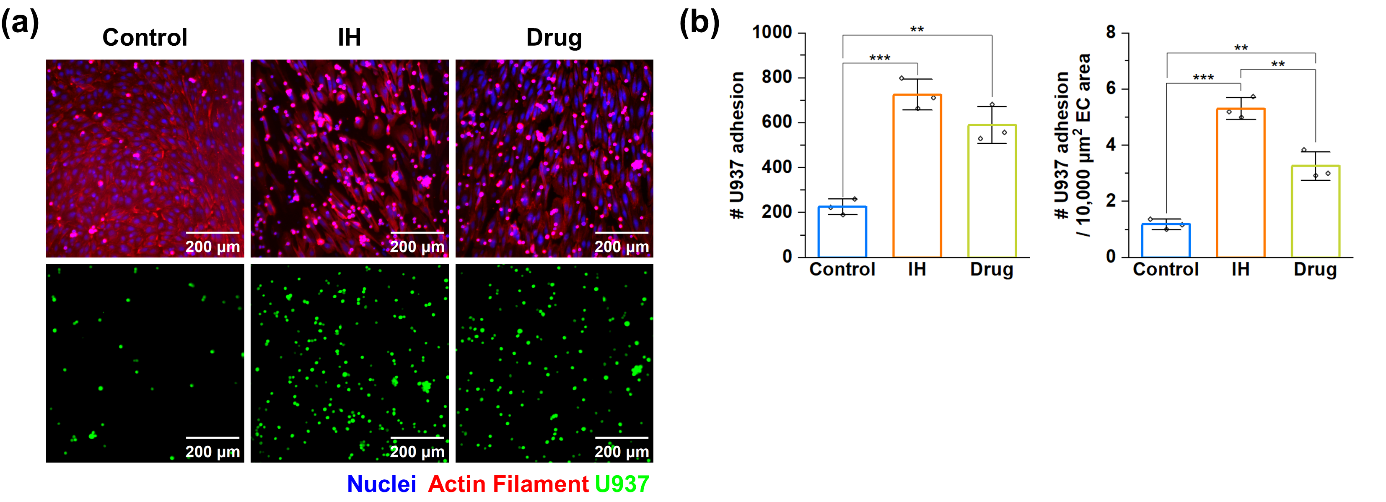


**Figure S9.** (a) Representative fluorescent images of immune cell adhesion to endothelium in each condition. (b) Quantification of the number of immune cell adhesion to endothelium (left) and the number of immune cell adhesion per 10,000 μm^2^ EC area (right). n=3 devices. Graphs represent the mean ± SD. Significance was determined using one-way ANOVA followed by Tukey’s post-hoc mean comparison between the two groups. ***p < 0.01*., ****p < 0.001*.


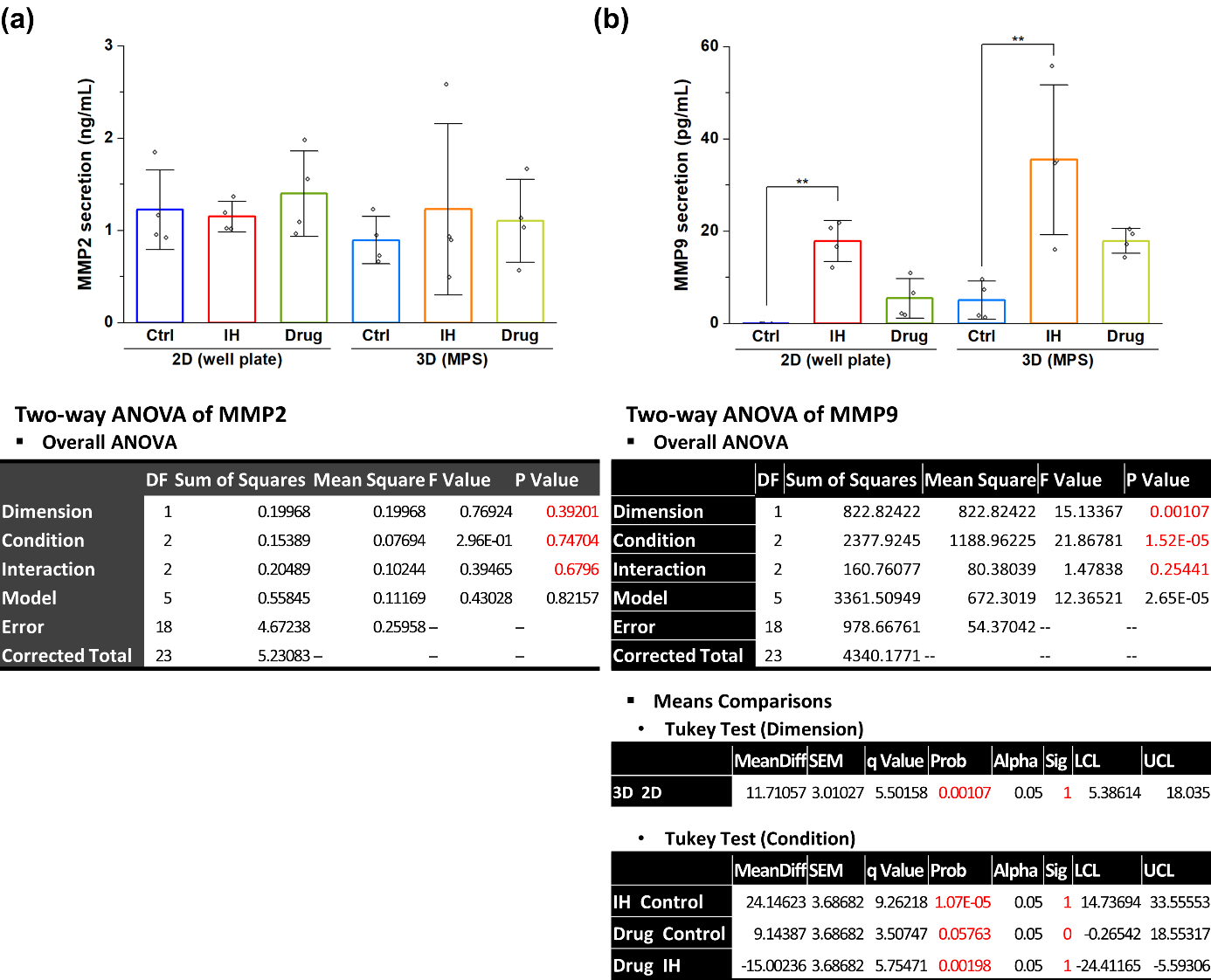


**Figure S10.** Measurement of MMPs using ELISA. (a) MMP2 secretion; (b) MMP9 secretion. n = 4. Graphs represent the mean ± SD. Significance was determined using two-way ANOVA followed by Tukey’s mean comparison between the two groups. Post hoc tests were conducted separately for 2D and 3D experiments. For 2D experiments, Dunn’s test was used due to zero average and variance in the 2D control group. For 3D experiments, Tukey’s test was applied. ***p < 0.01*.

**Table S1.** Concentration of drug solution

| Drug | Stock solution concentration  (solvent) | Final concentration in IH media |
| --- | --- | --- |
| Paclitaxel | 500 μM (DMSO) | 5 nM |
| Everolimus | 20 μM (DMSO) | 10 nM |
| Aspirin | 10 mM (EGM-2) | 3 mM |
| dexamethasone | 100 μM (DMSO) | 200 nM |
| resveratrol | 20 mM (DMSO) | 10 μM |
| DPI | 5 mM (DMSO) | 2.5 μM |
| quercetin | 50 mM (DMSO) | 20 μM |

**Table S2.** Reagents used for immunofluorescence staining.

| Antibody/probe | Species | Dilution ratio (v/v) | Vendor |
| --- | --- | --- | --- |
| VE-cadherin | Rabbit | 1:100 | Invitrogen |
| PECAM-1 (CD31) | Mouse | 1:300 | Abcam |
| ICAM-1 (CD 54) | Mouse | 1:100 | Invitrogen |
| vWF | Mouse | 1:100 | Invitrogen |
| Anti-rabbit Alexa fluor 488 | Goat | 1:200 | Invitrogen |
| Anti-mouse Alexa fluor 555 | Goat | 1:200 | Invitrogen |
| DAPI (4′,6-diamidino-2-phenylindole) | - | 1:1000 (1 μg/mL) | Invitrogen |
| rhodamine phalloidin | - | 1:400 (0.165 μM) | Invitrogen |
| Alexa fluor 647 phalloidin | - | 1:400 (0.165 μM) | Invitrogen |

**Table S3.** List of target genes.

| Gene symbol | Forward sequence | Reverse sequence | Product length (bp) |
| --- | --- | --- | --- |
| GAPDH | TCTGACTTCAACAGCGACAC | TGCTGTAGCCAAATTCGTTG | 115 |
| ANGPT1 | CTAGATTTCCAAAGAGGCTGG | GAATAGGCTCGGTTCCCTTC | 158 |
| ANGPT2 | AAAGTGGGATTTGGTAACCCT | GCACATAGCGTTGCTGATTA | 85 |
| NFE2L2 | TTGAGCAAGTTTGGGAGGAG | AGTTTGGCTTCTGGACTTGG | 109 |
| MMP2 | AAGGCTGTGTTCTTTGCAGG | CTCCAGTTAAAGGCGGCATC | 134 |
| vWF | CTCAAATACCTGTTCCCCGG | CTGCATCCCTTATTCCCCAC | 104 |
| CYBB | CTGTTCAATGCTTGTGGCTG | CTGCTCCCACTAACATCACC | 136 |
| NFKB1 | AGACATCCTTCCGCAAACTC | GGTCCTTCCTGCCCATAATC | 100 |
| STAT3 | AGGCGTCACTTTCACTTGG | GCTGCTTTGTGTATGGTTCC | 80 |
| VCAM1 | TGACCTTCATCCCTACCATTG | TGTATCTCTGGGGGCAACATT | 140 |
| PTGS2 | ATTCTTTGCCCAGCACTTCA | GTCTAGCCAGAGTTTCACCG | 125 |
| TAGLN | GGTGGAGTGGATCATAGTGC | ACCAGCTTGCTCAGAATCAC | 108 |
| MYH11 | AGTCCAAGTTCAAGTCCACC | TGTCTTTCTGCTTCAGCGAC | 120 |
| VIM | GGCACGTCTTGACCTTGAA | CCTGGATTTCCTCTTCGTGG | 86 |
| RUNX2 | TACTGTCATGGCGGGTAACG | GGTGAAACTCTTGCCTCGTC | 133 |
| BMP2 | GCAGCTTCCACCATGAAG | AGGTGATAAACTCCTCCGTG | 111 |

| Figure # | Statistical analysis | Post-hoc |
| --- | --- | --- |
| Figure 2b left panel | ANOVA | Tukey test |
| Figure 2b right panel | Welch’s ANOVA | Dunnett T3 test |
| Figure 2d left panel | ANOVA | Tukey test |
| Figure 2d right panel | ANOVA | Tukey test |
| Figure 3b upper panel | ANOVA | Tukey test |
| Figure 3b lower panel | ANOVA | Tukey test |
| Figure 3d left panel | ANOVA | Tukey test |
| Figure 3d right panel | Welch’s ANOVA | Games-Howell test |
| Figure 4 and 5 | unpaired two-tailed t-test | |
| Figure 6b upper panel | Mann-Whitney U test | |
| Figure 6b middle panel | unpaired two-tailed t-test | |
| Figure 6b lower panel | Mann-Whitney U test | |
| Figure 7 | unpaired two-tailed t-test | |
| Figure 8 ANGPT1 | ANOVA | Tukey test |
| Figure 8 ANGPT2 | ANOVA | Tukey test |
| Figure 8 VWF | ANOVA | Tukey test |
| Figure 8 NFE2L2 | ANOVA | Tukey test |
| Figure 8 MMP2 | ANOVA | Tukey test |
| Figure 8 STAT3 | ANOVA | Tukey test |
| Figure 8 NFKB1 | ANOVA | Tukey test |
| Figure 8 VCAM1 | ANOVA | Tukey test |
| Figure 8 PTGS2 | Welch’s ANOVA | Dunnett T3 test |
| Figure 8 TAGLN | ANOVA | Tukey test |
| Figure 8 VIM | ANOVA | Tukey test |
| Figure 8 BMP2 | ANOVA | Tukey test |


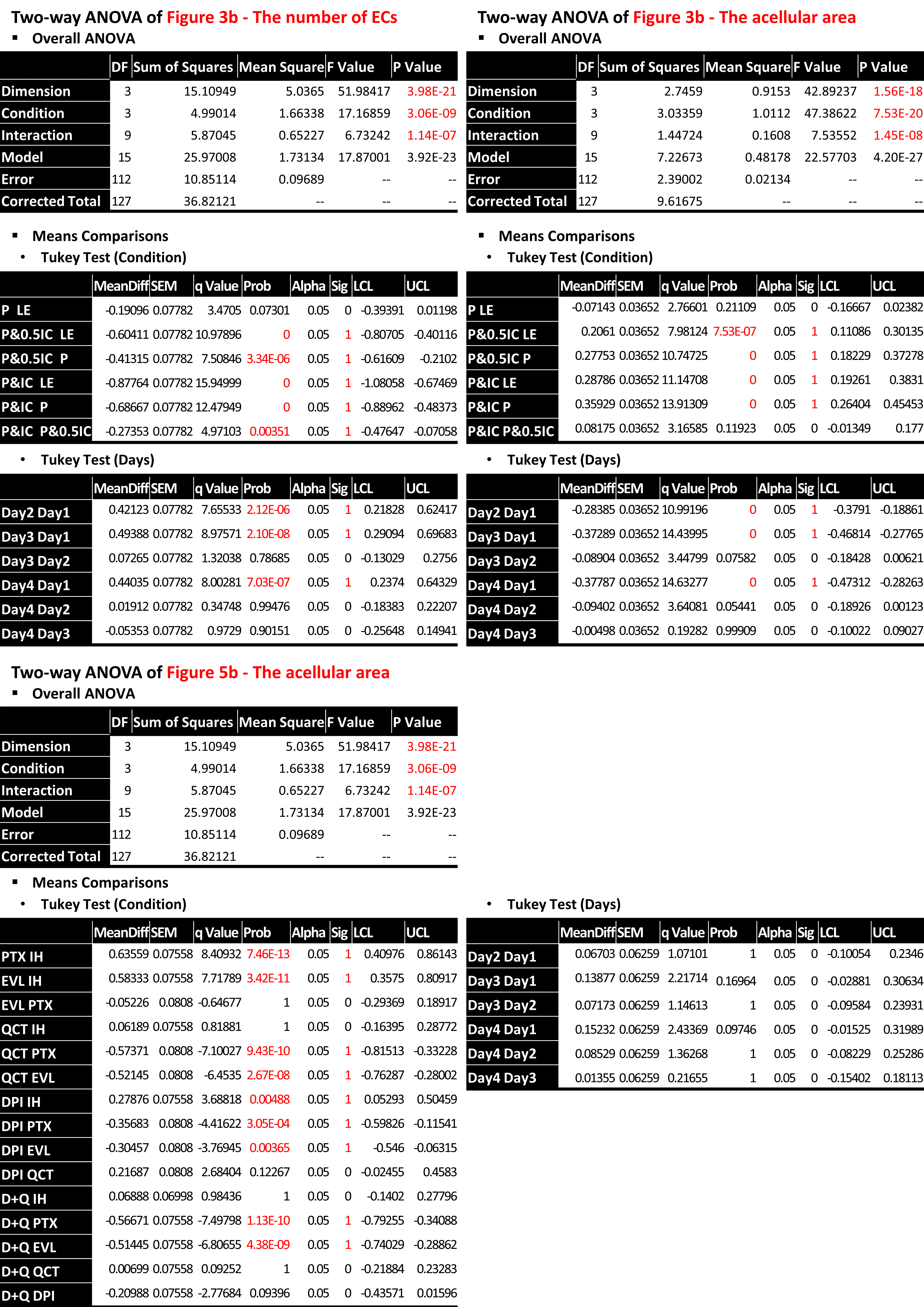


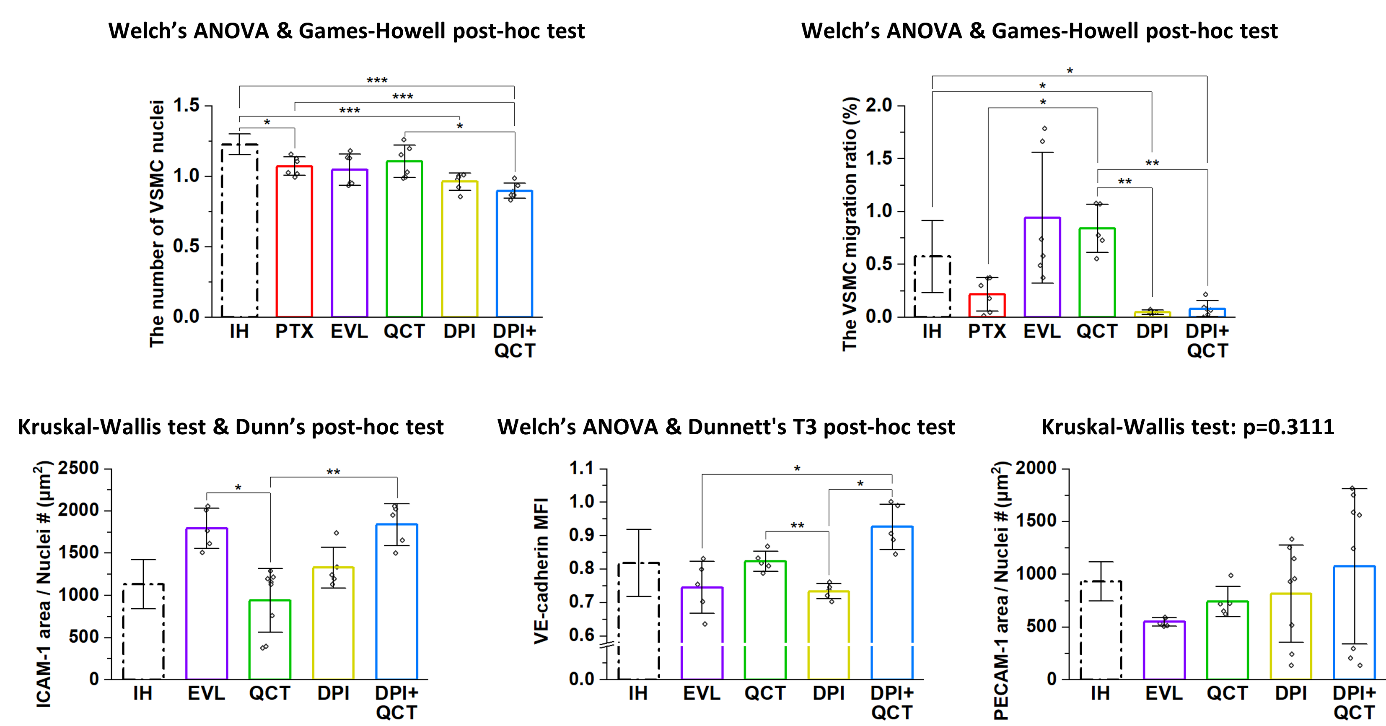

Supplement: Supplementary file 1 — Supporting Information [file ADHM-14-0-s001.docx]
